# Cancer-associated fibroblast-derived CXCL11 modulates hepatocellular carcinoma cell migration and tumor metastasis through the circUBAP2/miR-4756/IFIT1/3 axis

**DOI:** 10.1038/s41419-021-03545-7

**Published:** 2021-03-11

**Authors:** Gao Liu, Jian Sun, Zhang-Fu Yang, Cheng Zhou, Pei-Yun Zhou, Ruo-Yu Guan, Bao-Ye Sun, Zhu-Tao Wang, Jian Zhou, Jia Fan, Shuang-Jian Qiu, Yong Yi

**Affiliations:** Department of Liver Surgery and Transplantation, Liver Cancer Institute and Biomedical Research Center, Zhongshan Hospital, Fudan University, Shanghai, 200032 PR China

**Keywords:** Tumour biomarkers, Cancer

## Abstract

Cancer-associated fibroblasts (CAFs) are commonly acquired activated extracellular matrix (ECM)-producing myofibroblasts, a phenotypes with multiple roles in hepatic fibrogenesis and carcinogenesis via crosstalk with cohabitating stromal/cancer cells. Here, we discovered a mechanism whereby CAF-derived cytokines enhance hepatocellular carcinoma (HCC) progression and metastasis by activating the circRNA-miRNA-mRNA axis in tumor cells. CAFs secreted significantly higher levels of CXCL11 than normal fibroblasts (NFs), and CXCL11 also had comparatively higher expressions in HCC tissues, particularly in metastatic tissues, than para-carcinoma tissues. Both CAF-derived and experimentally introduced CXCL11 promoted HCC cell migration. Likewise, CAFs promoted tumor migration in orthotopic models, as shown by an increased number of tumor nodules, whereas CXCL11 silencing triggered a decrease of it. CXCL11 stimulation upregulated circUBAP2 expression, which was significantly higher in HCC tissues than para-carcinoma tissues. Silencing circUBAP2 reversed the effects of CXCL11 on the expression of IL-1β/IL-17 and HCC cell migration. Further downstream, the IFIT1 and IFIT3 levels were significantly upregulated in HCC cells upon CXCL11 stimulation, but downregulated upon circUBAP2 silencing. IFIT1 or IFIT3 silencing reduced the expression of IL-17 and IL-1β, and attenuated the migration capability of HCC cells. Herein, circUBAP2 counteracted miR-4756-mediated inhibition on IFIT1/3 via sponging miR-4756. miR-4756 inhibition reversed the effects induced by circUBAP2 silencing on the IL-17 and IL-1β levels and HCC cell migration. In orthotopic models, miR-4756 inhibition also reversed the effects on metastatic progression induced by silencing circUBAP2.

## Introduction

The occurrence of intrahepatic or systemic metastasis is the main cause that result in poor prognosis of patients with advanced HCC^1^. Lung metastasis is the most common outcome of progression and one of the leading causes of cancer deaths caused by HCC^2,3^. Approximately 80–90% of HCC develops with cirrhosis or fibrosis as a required intermediate^4^, and approximately one-third of cirrhosis patients have a lifetime risk of developing HCC^5^. Further elucidation on the mechanisms by which myofibroblasts integrate with their cohabitating cancer cells and how they bridge fibrosis and HCC through microenvironment remodeling might lead to the development of anticancer strategies.

Reportedly, cancer-associated fibroblasts (CAFs) originate from activated hepatic stellate cells and progressively evolve into a major source of ECM as a consequence of their accumulation in the stroma of HCC^6–10^. Increasing data now show that CAFs exert protumorigenic effects in tumor proliferation, migration, invasion, and EMT (epithelial–mesenchymal transformation) through crosstalk with cancer cells via products of their paracrine repertoire^11,12^. Over the last decade, a diverse range of CAFs-derived chemokines, such as CCL2, CCL26, IL6, CXCL1, and CXCL8, has been reported to be mechanistically related to tumor progression^13,14^. In addition, other mediators including IL-1, CXC, and CC chemokines are also involved^15,16^. These factors might be the mediators of CAFs contributing to carcinogenesis.

Recently, mounting evidence has shown that circRNAs play a mediating role in tumor biology through various mechanisms related to the modulatory interactions among circRNAs, miRNAs, and mRNA^17,18^. An increasing number of studies have noted that circRNA act as competing endogenous RNA (ceRNA) and subsequently counteract miRNA-induced effects on downstream mRNAs by sponging miRNAs^19^. For example, circRNA 001306 promoted HCC progression by upregulating CK16 by sponging miR-584-5p^20^. In addition, miRNAs, such as miR-101 and miR-346, have been demonstrated to play crucial roles in CAF-directed HCC progression^21,22^. However, it has not yet been fully determined how the circRNA-miRNA-mRNA network functions under the effects of CAFs during HCC progression.

Given the roles of CAF-derived chemokines and noncoding RNAs in tumor progression and metastasis, we hypothesized that tumor progression directed by CAF-derived chemokines engages certain circRNA-miRNA-mRNA axes in tumor cells. Herein, we show that CAF-derived CXCL11 was the most upregulated candidate among the 17 differentially expressed chemokine candidates, and that elevation of CXCL11 in tumor microenvironment promoted hepatocellular carcinoma cell migration and tumor metastasis through the circUBAP2/miR-4756/IFIT1/3 axis both in vivo and in vitro.

## Results

### CXCL11 expression in tissue samples and cancer-associated fibroblasts (CAFs)

**Fig. 1:**
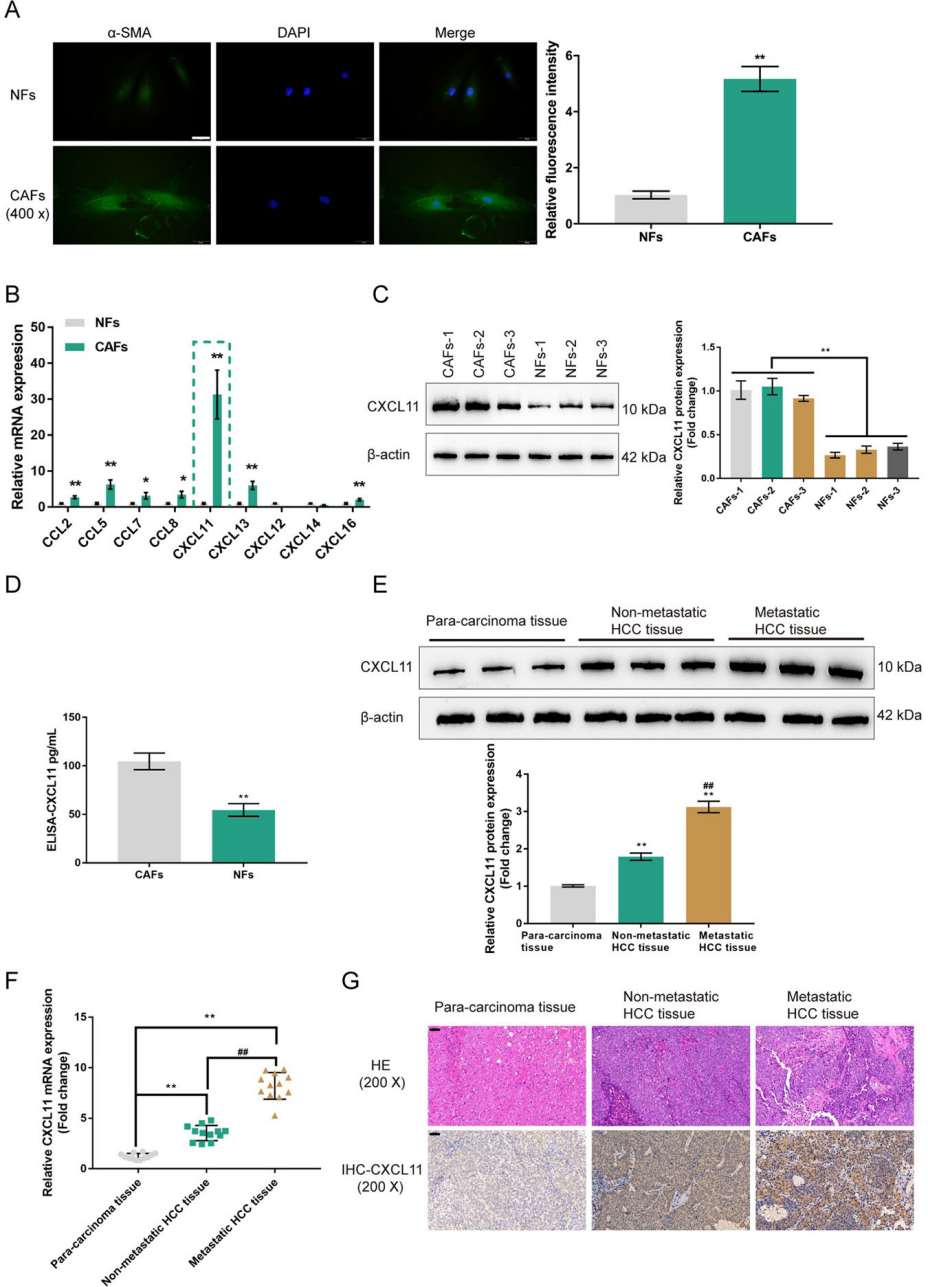
Expression of CXCL11 in tissue samples and cancer-associated fibroblasts (CAFs). **A** CAFs and normal fibroblasts were isolated from cancer tissues and noncancerous normal tissues, respectively, and identified for α-SMA protein content and distribution by Immunofluorescent (IF) staining. The relative fluorescence intensity was shown in the right panel. *N* = 5. **B** The mRNA expression of CXCL13, CXCL12, CCL2, CXCL11, CXCL16, CCL8, CXCL26, CXCL5, CXCL14, CCL5, and CCL7 was examined in CAFs and NFs using real-time PCR. **C** The protein levels of CXCL11 were examined in CAFs and NFs using Immunoblotting. **D** The contents of CXCL11 in the culture medium were determined by ELISA. **E** The protein levels of CXCL11 were examined in 12 cases of para-carcinoma tissues, 12 cases of nonmetastatic HCC tissues, and 12 cases of metastatic HCC tissues using Immunoblotting. **F** The mRNA expression of CXCL11 was examined in 12 cases of para-carcinoma tissues, 12 cases of nonmetastatic HCC tissues, and 12 cases of metastatic HCC tissues using real-time PCR. **G** The histopathological features of para-carcinoma tissues, nonmetastatic HCC tissues, and metastatic HCC tissues were examined by H&E staining. The protein contents of CXCL11 in para-carcinoma tissues, nonmetastatic HCC tissues, and metastatic HCC tissues were examined by Immunohistochemical (IHC) staining. *P < 0.05, **P < 0.01, compared with NFs group or para-carcinoma tissue group; ^##^*P* < 0.01, compared with the nonmetastatic HCC tissue group.

CAFs and NFs were isolated from cancer tissues and noncancerous normal tissues, respectively. As shown in Fig. 1A, relative fluorescence intensity of green fluorescence representing the cytoskeletal protein α-SMA was dramatically enhanced in CAFs compared with NFs. Previous studies reported, CAFs secreted higher levels of CCL2, CCL5, CCL7, CXCL16^12^, CCL8^23^, CXCL11^24^, CXCL12^25^, CXCL13^26^, and CXCL14 than NFs^27^. Besides, both GSE14323 and GSE6764 indicated that these chemokines were highly expressed in HCV-induced liver cirrhosis or liver cancer tissues. Therefore, we examined the mRNA expression of CCL2, CCL5, CCL7, CCL8, CXCL11, CXCL12, CXCL13, CXCL14, and CXCL16 in CAFs and NFs. We found that the expression of CXCL11 was most dramatically upregulated in CAFs when compared with NFs (Fig. 1B). Additionally, CXCL11 expression was also upregulated in liver cirrhosis or liver cancer tissues, compared to normal liver tissues, within the GSE14323 and GSE6764 datasets (Fig. S1A, B).

The intracellular and secreted levels of CXCL11 in CAFs and NFs were also examined. The CXCL11 protein levels were significantly upregulated in CAFs compared with NFs (Fig. 1C). The secreted CXCL11 level was also higher in CAF culture medium than that in NF culture medium (Fig. 1D). Consistently, the protein level of CXCL11 was increased in the nonmetastatic HCC tissue samples and the metastatic HCC tissue samples compared with the in para-carcinoma tissues, and was further increased in the metastatic HCC tissue samples compared with the nonmetastatic HCC tissue samples (Fig. 1E). Besides, the CXCL11 mRNA levels were increased in the nonmetastatic HCC tissue samples and metastatic HCC tissue samples compared with the para-carcinoma tissue samples and were greater in the metastatic HCC tissue samples than in the nonmetastatic HCC tissue samples (Fig. 1F). With anti-CXCL11, similar results were observed in IHC staining, in which CXCL11 proteins were significantly increased in nonmetastatic HCC tissue samples and metastatic HCC tissue samples, and greater in the metastatic HCC tissues than the nonmetastatic HCC tissues (Fig. 1G).

### CAFs-derived CXCL11 affects the HCC cell phenotype

To validate the specific roles of CAF-derived CXCL11 in the malignant behaviors of HCC cell, we collected different conditioned media (CMs) for the HCC cell lines (MHCC-97H and Huh-7): control medium (control), NFs-derived conditioned medium (NFs-CM), CAFs-derived conditioned medium (CAFs-CM), CAFs-CM supplemented with PBS (CAFs-CM/PBS), CAFs-CM supplemented with 5 ng/ml CXCL11 (CAFs-CM/CXCL11 (5 ng/ml)), CAFs-CM supplemented with 10 ng/ml CXCL11 (CAFs-CM/CXCL11 (10 ng/ml)), CAFs-CM supplemented with 1 μg/ml IgG (CAFs-CM/IgG), and CAFs-CM supplemented with 1 μg/ml CXCL11 neutralizing antibody (CAFs-CM/anti-CXCL11).

**Fig. 2:**
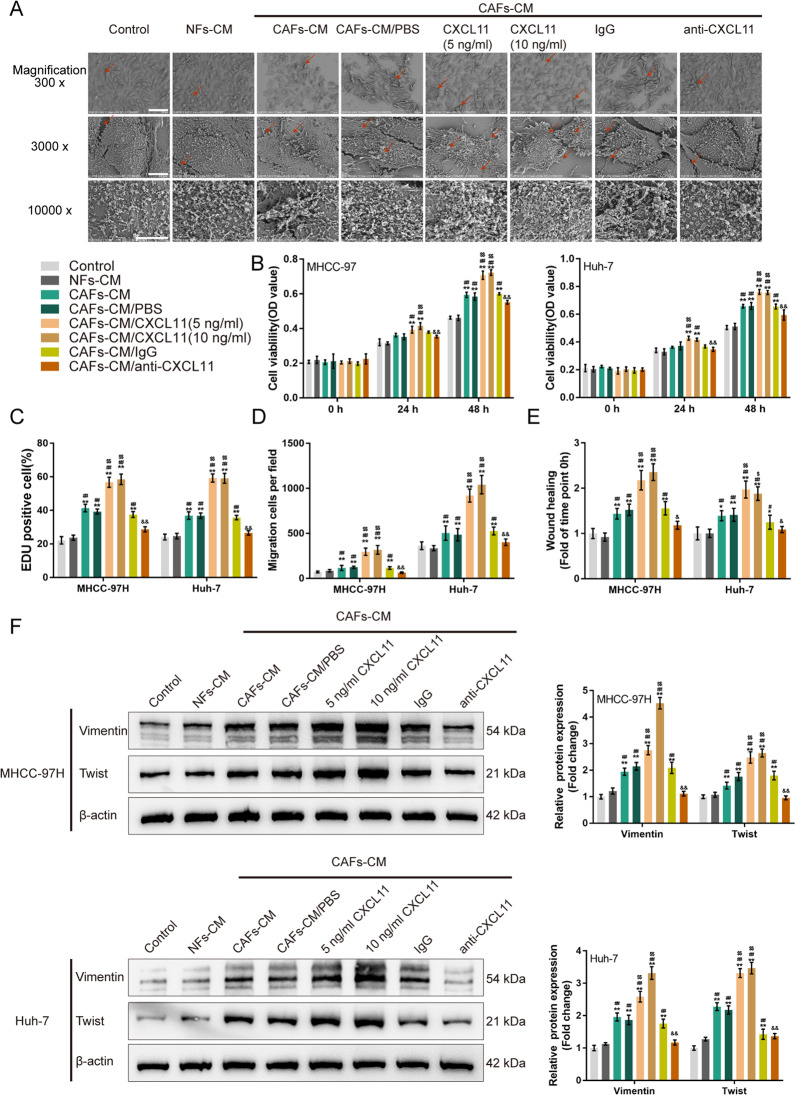
CAFs-derived CXCL11 affecting hepatocellular carcinoma (HCC) cell phenotype. HCC cell lines, MHCC-97H, and Huh-7, were cultured in control medium (Control), NFs-derived conditioned medium (NFs-CM), CAFs-derived conditioned medium (CAFs-CM), CAFs-CM added with PBS (CAFs-CM/PBS), CAFs-CM added with 5 ng/ml CXCL11 (CAFs-CM/CXCL11 (5 ng/ml)), CAFs-CM added with 10 ng/ml CXCL11 (CAFs-CM/CXCL11 (10 ng/ml)), CAFs-CM added with IgG (CAFs-CM/IgG), and CAFs-CM added with anti-CXCL11 (CAFs-CM/anti-CXCL11). **A** The morphological changes of HCC cell line MHCC-97H were examined by scanning electron microscopy (SEM) with different magnifications; red arrows indicated the pseudopodia of HCC cells. **B** HCC cells viability was determined by MTT assays. **C** HCC cell DNA synthesis ability was determined by EDU assay. **D** HCC cell migration was examined using Transwell assay. **E** HCC cell migration was examined by wound healing assay. **F** The protein levels of Vimentin and Twist was examined by Immunoblotting. *P < 0.05, **P < 0.01, compared with the control group; ^#^*P* < 0.05, ^##^*P* < 0.01, compared with the NFs-CM group; ^$^*P* < 0.05, ^$$^*P* < 0.01, compared with the CAFs-CM/*P*BS group; ^&^*P* < 0.05, ^&&^*P* < 0.01, compared with the CAFs-CM/IgG group.

Then, MHCC-97H and Huh-7 cells were cultured in different media accordingly and examined for morphological changes and cell migration. Under SEM using different magnifications, MHCC-97H cells in the CAFs-CM, CAFs-CM/PBS, CAFs-CM/CXCL11 (5 ng/ml), CAFs-CM/CXCL11 (10 ng/ml), and CAFs-CM/IgG groups changed from flat-shaped to spindle-shaped, and the pseudopodia increased (Fig. 2A), but the appearance of HCC cells in NFs-CM and CAFs-CM/anti-CXCL11 showed no significant changes compared to that of the control (Fig. 2A).

Next, HCC cell proliferation and migration in the different medium was examined. As shown in Fig. 2B–E and Fig. S2A, B, compared to those of the control and NFs-CM groups, proliferation, DNA synthesis and migration of HCC cells was significantly augmented in CAFs-CM, CAFs-CM/PBS, CAFs-CM/CXCL11 (5 ng/ml), CAFs-CM/CXCL11 (10 ng/ml), and CAFs-CM/IgG groups (*P < 0.05, **P < 0.01, ^#^*P* < 0.05, ^##^*P* < 0.01), but these showed no significant changes in CAFs-CM/anti-CXCL11 group. Compared with those of CAFs-CM and CAFs-CM/PBS groups, HCC cell proliferation, DNA synthesis and migration ability were significantly promoted in CAFs-CM/CXCL11 (5 ng/ml) and CAFs-CM/CXCL11 (10 ng/ml) groups (^$^*P* < 0.05, ^$$^*P* < 0.01; Fig. 2B–E). Compared with those of CAFs-CM/IgG group, HCC cell proliferation, DNA synthesis and migration were significantly inhibited in CAFs-CM/anti-CXCL11 group (^&^*P* < 0.05, ^&&^*P* < 0.01; Fig. 2B–E).

We then examined the changes in the Vimentin and Twist protein levels in different groups in search of bio-factors related to tumor migration. Compared to those of the control and NFs-CM groups, Vimentin and Twist were dramatically increased in CAFs-CM, CAFs-CM/PBS, CAFs-CM/CXCL11 (5 ng/ml), CAFs-CM/CXCL11 (10 ng/ml), and CAFs-CM/IgG groups (*P < 0.05, **P < 0.01, ^#^*P* < 0.05, ^##^*P* < 0.01; Fig. 2E and Fig. S2C), but Vimentin and Twist in the CAFs-CM/anti-CXCL11 group showed no significant changes. And, compared with those of the CAFs-CM and CAFs-CM/PBS groups, Vimentin and Twist were significantly increased in the CAFs-CM/CXCL11 (5 ng/ml) and CAFs-CM/CXCL11 (10 ng/ml) groups (^$^*P* < 0.05, ^$$^*P* < 0.01; Fig. 2E and Fig. S2C). Compared with those of the CAFs-CM/IgG group, Vimentin and Twist were significantly decreased in the CAFs-CM/anti-CXCL11 group (^&^*P* < 0.05, ^&&^*P* < 0.01; Fig. 2E and Fig. S2C).

### CAFs-derived CXCL11 affects the tumor growth in BALB/c nude mice with orthotopically implanted tumors

To investigate the in vivo effects of CAFs-derived CXCL11, we first generated orthotopic implantation tumor models in mice. Mice were randomly assigned to five groups: the Huh-7 cells group (*n* = 6), the Huh-7 cells + NFs group (*n* = 6), the Huh-7 cells + CAFs (untransfected; *n* = 6) group, the Huh-7 cells + CAFs (transfected with sh-NC; *n* = 6) group, and the Huh-7 cells + CAFs (transfected with sh-CXCL11; *n* = 6) group. Fourteen days after cell injection, the mice were then anesthetized and sacrificed, the livers were collected (Fig. S3A), and the tumor nodules were counted. As shown in Fig. S3B, there were more tumor nodules in the Huh-7 cells + CAFs and Huh-7 cells + CAFs (sh-NC) groups than that in the Huh-7 cells and Huh-7 cells + NFs groups; there were fewer tumor nodules in the Huh-7 cells + CAFs group than that in the Huh-7 cells + CAFs and Huh-7 cells + CAFs (sh-NC) groups. Livers from different groups were evaluated by H&E staining for histopathological characteristics (Fig. S3C). In orthotopic tumor tissues, CXCL11 protein expression was dramatically higher in the Huh-7 cells + CAFs and Huh-7 cells + CAFs (sh-NC) groups compared with those in the Huh-7 cells + CAFs and Huh-7 cells + CAFs (sh-NC) groups (Fig. S3D, E).

### Bioinformatic analysis of circRNAs related to the functions of CAF-secreted CXCL11 in aggressive HCC

**Fig. 3:**
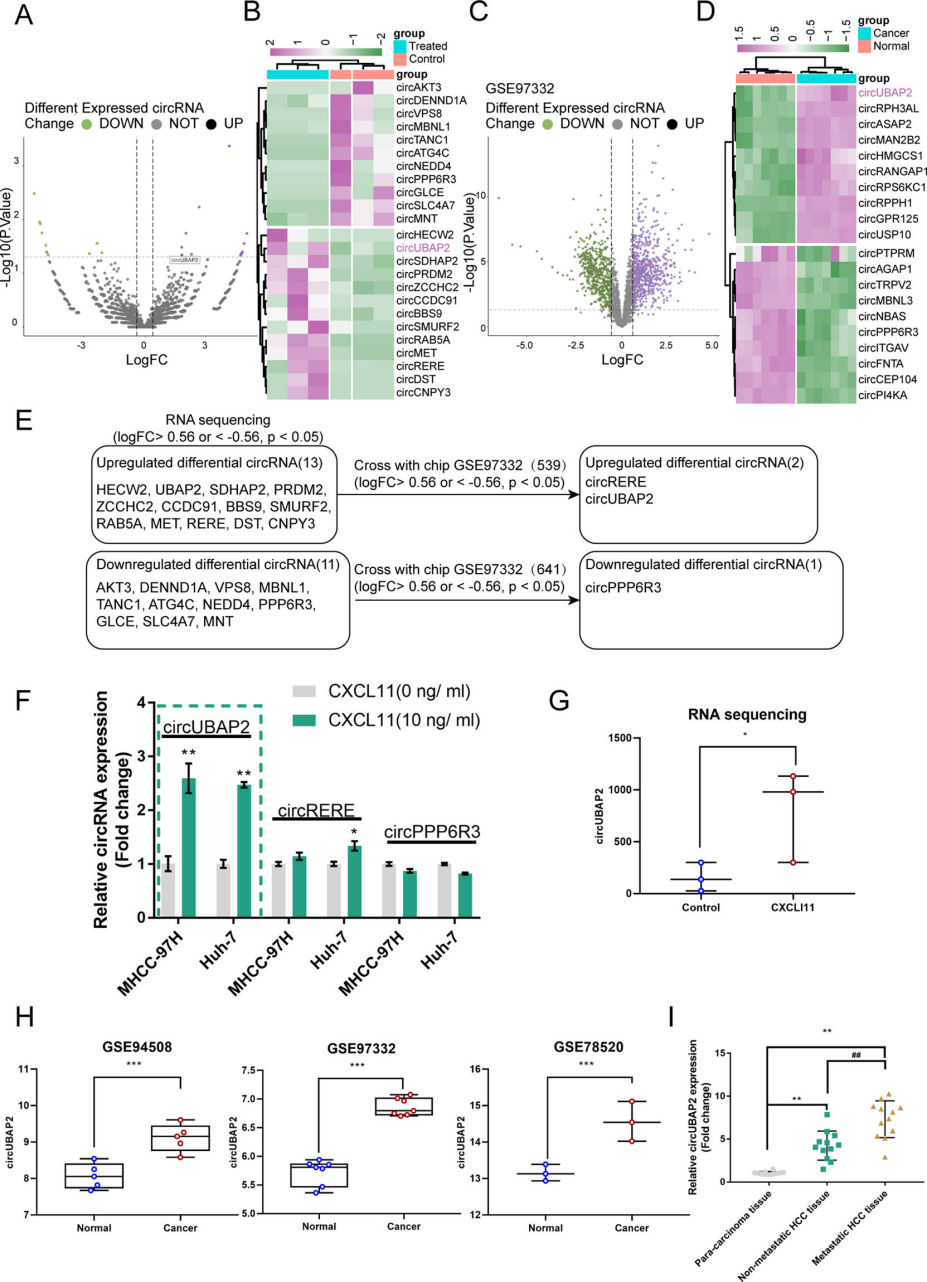
Selection of circRNA related to the functions of CAF-secreted CXCL11 on HCC cell aggressiveness. **A**, **B** Volcano plot and hierarchical clustering heatmap showing differentially expressed circRNAs in CXCL11-treated MHCC-97H based on RNA sequencing. **C**, **D** Volcano plot and hierarchical clustering heatmap showing differentially expressed circRNAs between HCC and normal tissues based on GSE97332. **E** A schematic diagram showing the selection of circRNAs related to the functions of CAF-secreted CXCL11 on HCC cell aggressiveness based on our RNA sequencing data and online microarray expression profiles. These datasets intersected at the following circRNAs: circUBAP2 (upregulated), circRERE (upregulated), and circPPP6R3 (downregulated). **F** HCC cell lines MHCC-97H and Huh-7 were treated with CXCL11 (10 ng/ml) and examined for the expression of circUBAP2, circRERE, and circPPP6R3 by real-time PCR. **G** The expression of circUBAP2 in CXCL11-treated or untreated MHCC-97H and Huh-7 cells. **H** The expression of circUBAP2 in normal liver tissues and liver cancer tissues based on GSE97332, GSE94508 and GSE78520. **I** The expression of circUBAP2 was examined in 12 cases of nonmetastatic HCC tissues, metastatic HCC tissues, and para-carcinoma tissues by real-time PCR. **P < 0.01, ^##^*P* < 0.01.

We performed RNA sequencing on HCC cells with/without CXCL11 to validate the possibility that circRNAs contribute to the oncogenic effects of CAF-secreted CXCL11 in aggressive HCC. A total of 3124 circRNAs were detected and 24 were significantly differentially expressed (|logFC| > 0.56, *p* < 0.05); 11 were downregulated and 13 were upregulated (Fig. 3A, B). According to GSE97332, a total of 1180 circRNAs exhibited differential expression within HCC tissue samples compared with normal liver tissue samples; 641 were upregulated and 539 were downregulated (Fig. 3C). The top 10 upregulated and downregulated circRNAs were shown in Fig. 3D. The intersection of RNA sequencing data and GSE97332 included circRERE and circUBAP2 (upregulated) and circPPP6R3 (downregulated), and according to all four datasets, circUBAP2 was significantly upregulated in the CXCL11-treated HCC cells or HCC tissues (Fig. 3E).

Next, we treated the HCC cell lines MHCC-97H and Huh-7 with CXCL11 (10 ng/ml) and performed real-time PCR to determine the circRERE, circUBAP2, and circPPP6R3 expression levels. Figure 3F shows that only circUBAP2 expression was dramatically induced by CXCL11 stimulation. The expression of circUBAP2 was significantly upregulated in the CXCL11-treated MHCC-97H cells compared with the untreated MHCC-97H cells (Fig. 3G) and increased in the HCC tissue samples compared with the noncancerous tissue samples according to GSE94508, GSE97332, and GSE78520 (Fig. 3H). Moreover, circUBAP2 expression was significantly increased in the nonmetastatic HCC tissue samples and metastatic HCC tissue samples compared with the para-carcinoma tissue samples and was increased in the metastatic HCC tissue samples than that within nonmetastatic HCC tissue samples (Fig. 3I). Thus, circUBAP2 was chosen for further experiments.

### circUBAP2 is involved in the effects of CXCL11 on HCC cells

**Fig. 4:**
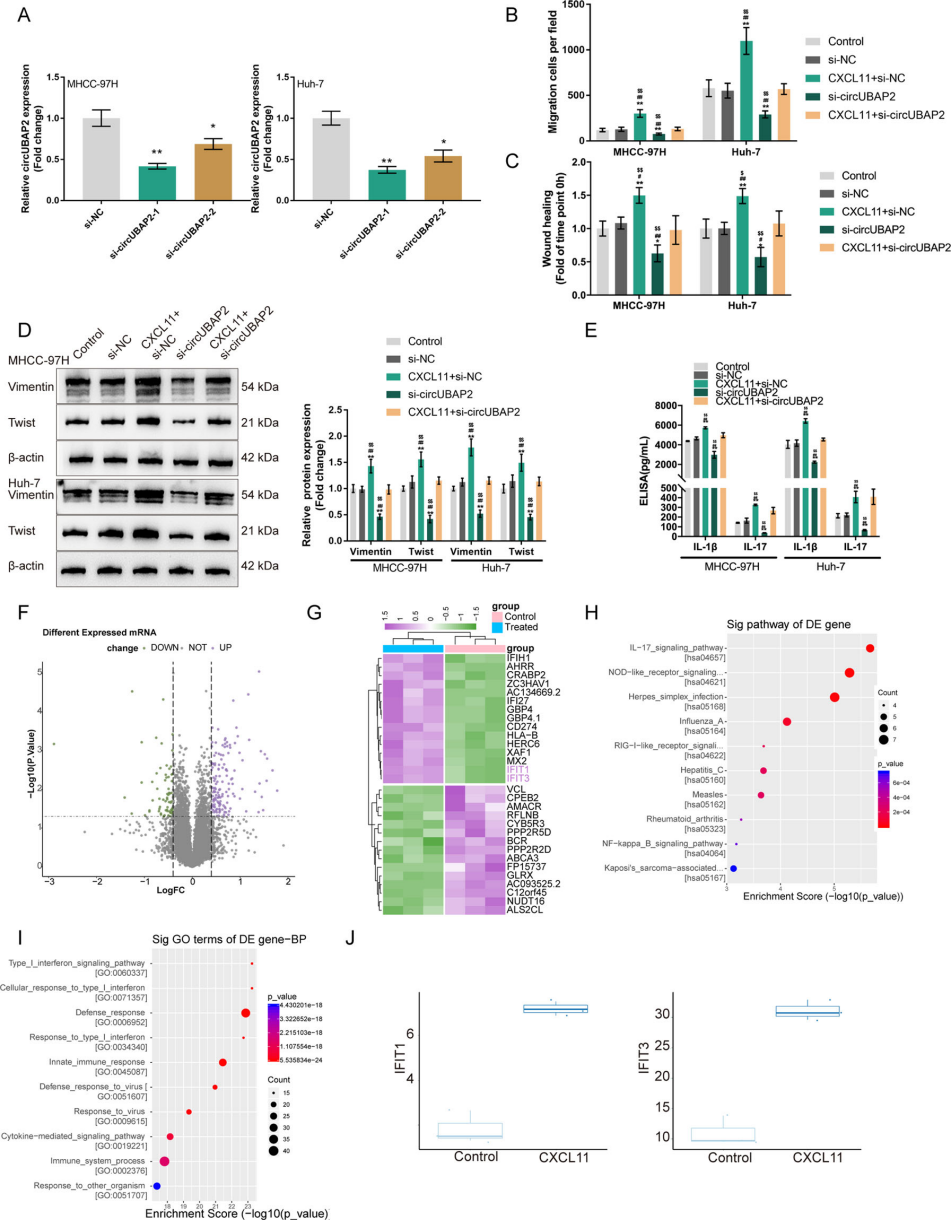
The function of circUBAP2 in the effects of CAFs-derived CXCL11 on HCC cells and the selection of mRNA related to the functions of circUBAP2. **A** Silencing of circUBAP2 was achieved in MHCC-97H and Huh-7 cells by transfecting small interfering RNA targeting circUBAP2 (si-circUBAP2-1 or si-circUBAP2-2). Si-NC was transfected as a negative control. The transfection efficiency was confirmed by real-time PCR and si-circUBAP2-1 was chosen for further experiments for its better transfection efficiency. Then, MHCC-97H and Huh-7 cells were transfected with si-circUBAP2 and examined for **B** Cell migration by Transwell assay; **C** Cell migration by wound healing assay; **D** The protein levels of Vimentin and Twist was examined by Immunoblotting; **E** The concentrations of IL-1β and IL-17 in the culture medium by ELISA. **F**, **G** The Volcano plot and hierarchical clustering heatmap showing differentially expressed mRNAs in CXCL11-treated MHCC-97H based on RNA sequencing. Upregulated genes were applied for **H** Kyoto Encyclopedia of Genes and Genomes (KEGG) signaling enrichment analysis and **I** Gene Ontology (GO) of biological process enrichment analysis. **J** The expression of IFIT1/IFIT3 in CXCL11-treated and untreated HCC cells based on RNA sequencing data. *P < 0.05, **P < 0.01, compared with the control group; ^#^*P* < 0.05, ^##^*P* < 0.01, compared with the si-NC group; ^$^*P* < 0.05, ^$$^*P* < 0.01, compared with the CXCL11 + si-circUBAP2 group.

Since CXCL11 significantly induces circUBAP2 expression, we achieved the silencing of circUBAP2 in MHCC-97H and Huh-7 cells by transfecting siRNA targeting circUBAP2 (si-circUBAP2-1/si-circUBAP2-2); si-NC was transfected as a negative control, as confirmed by real-time PCR; and si-circUBAP2-1 was chosen for further experiments because it had better transfection efficiency than the other siRNA (Fig. 4A). Then, we divided MHCC-97H and Huh-7 cells into five groups: the control group (untreated, untransfected), si-NC group (untreated, transfected with si-NC), CXCL11 + si-NC group (treated with 10 ng/ml CXCL11, transfected with si-NC), si-circUBAP2 group (untreated, transfected with si-circUBAP2), and CXCL11 + si-circUBAP2 group (treated with 10 ng/ml CXCL11, transfected with si-circUBAP2). HCC cells were examined for cell migration and related markers. Compared to that of the control and si-NC groups, the migratory ability of HCC cells was significantly promoted in the CXCL11 + si-NC group but inhibited in the si-circUBAP2 group; the promotive effects of CXCL11 stimulation on HCC cell migration were significantly reversed by circUBAP2 silencing (Fig. 4B, C and Fig. S4A, B). For the cell migration-related markers, compared with those in the control and si-NC groups, the Vimentin and Twist protein levels were dramatically increased in the CXCL11 + si-NC group but decreased in the si-circUBAP2 group; the effects of CXCL11 stimulation on the Vimentin and Twist protein levels were partially reversed by circUBAP2 silencing (Fig. 4D and Fig. S4C). Moreover, the concentrations of the IL-17 signaling cytokines, IL-1β and IL-17, were examined. Similarly, compared with those in the control and si-NC groups, the medium concentrations of IL-1β and IL-17 were dramatically higher in the CXCL11 + si-NC group but lower in the si-circUBAP2 group; the effects of CXCL11 stimulation on the IL-1β and IL-17 medium concentrations were partially reversed by circUBAP2 silencing (Fig. 4E).

### Bioinformatic analysis of mRNAs related to the functions of CAF-secreted CXCL11 and circUBAP2 in aggressive HCC

RNA-Seq was performed on CXCL11-stimulated or unstimulated HCC cells to identify differentially expressed genes. According to our RNA sequencing data, a total of 11622 mRNAs were detected and 211 mRNAs exhibited significant differential expression; of them, 142 were increased and 69 were decreased in HCC cells via CXCL11 stimulation (Fig. 4F). The top 15 upregulated or downregulated genes were shown in the clustered heat map (Fig. 4G). These upregulated genes were subjected to KEGG signaling enrichment analysis and Gene GO enrichment analysis. As shown in Fig. 4H, KEGG enrichment annotation (https://www.genome.jp/kegg/) suggested that these upregulated genes were significantly enriched in IL-17 signaling, Nod-like receptor signaling, herpes simplex infection pathway and so on (Table S2). GO enrichment analysis of the biological process also suggested that these upregulated genes were obviously enriched signaling related to the cellular response to type I interferon (Fig. 4I). Among these differentially expressed genes, interferon-induced protein with tetratricopeptide repeats 1 (IFIT1) and IFIT3 are interferon signaling related genes^28^ and were both significantly upregulated in HCC cells by CXCL11 stimulation (Fig. 4J). Thus, we speculated that IFIT1/IFIT3 might participate in the functions of CAF-secreted CXCL11 and circUBAP2 in HCC cells.

### Effects of IFIT1/IFIT3 on HCC cell phenotype

**Fig. 5:**
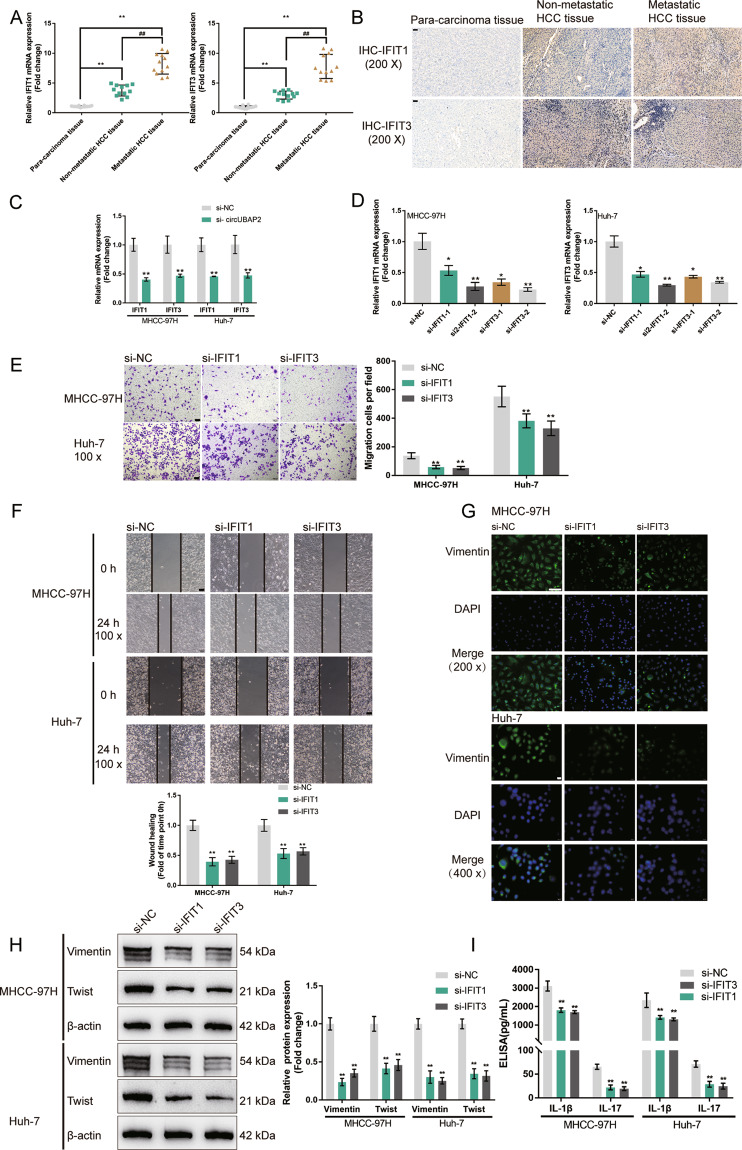
Effects of IFIT1/IFIT3 on HCC cell phenotype. **A** The mRNA expression of IFIT1 and IFIT3 was examined in 12 cases of nonmetastatic HCC tissues, metastatic HCC tissues, and para-carcinoma tissues by real-time PCR. **B** The protein contents and distribution of IFIT1 and IFIT3 were examined in nonmetastatic HCC tissues, metastatic HCC tissues, and para-carcinoma tissues by IHC staining. **C** MHCC-97H and Huh-7 cells were transfected with si-circUBAP2 and examined for the mRNA expression of IFIT1 and IFIT3 by real-time PCR. **D** IFIT1 or IFIT3 silencing was achieved in MHCC-97H and Huh-7 cells by transfecting small interfering RNA targeting IFIT1 or IFIT3 (si-IFIT1-1 or si-IFIT1-2; si-IFIT3-1 or si-IFIT3-2). Si-NC was transfected as a negative control. The transfection efficiency was confirmed by real-time PCR and si-IFIT3-1 and si-IFIT1-2 were chosen for further experiments due to better transfection efficiency. Next, MHCC-97H and Huh-7 cells were transfected with si-NC, si-IFIT1, or si-IFIT3, and examined for **E** Cell migration by Transwell assay; **F** Cell migration by wound healing assay; **G** Cellular protein content and distribution of Vimentin was examined by IF staining; **H** The protein levels of Vimentin and Twist was examined by Immunoblotting; **I** The concentrations of IL-1β and IL-17 in the culture medium by ELISA. *P < 0.05, **P < 0.01, compared with the si-NC group.

Firstly, we determined the expression of IFIT1 and IFIT3 in tissues. We found that the mRNA levels of IFIT1 and IFIT3 were dramatically increased in the nonmetastatic HCC tissue samples and the metastatic HCC tissue samples compared with the para-carcinoma tissue samples, and increased in the metastatic HCC tissue samples compared with the nonmetastatic HCC tissue samples (Fig. 5A). The protein levels of IFIT1 and IFIT3 showed a similar expression trend as their mRNA levels in HCC tissues (Fig. 5B). Figure 5C shows that circUBAP2 knockdown dramatically downregulated IFIT1 and IFIT3 mRNA expression in MHCC-97H and Huh-7 cells.

Next, we transfected small interfering RNA targeting IFIT1 or IFIT3 to achieve IFIT1 or IFIT3 silencing in MHCC-97H and Huh-7 cells. As confirmed by real-time PCR, si-IFIT3-1 and si-IFIT1-2 were chosen for further experiments due to their best transfection efficiency (Fig. 5D). As revealed by Transwell and wound healing assays, IFIT1 silencing or IFIT3 silencing was sufficient to inhibit HCC cell migration (Fig. 5E, F). Consistently, IF staining and Immunoblotting also showed that IFIT1 silencing or IFIT3 silencing was sufficient to decrease the expressions of Vimentin and Twist (Fig. 5G, H). Moreover, IFIT1 silencing or IFIT3 silencing significantly reduced the medium concentrations of IL-17 and IL-1β (Fig. 5I). These data indicated that IFIT1 silencing or IFIT3 silencing attenuates the HCC cell aggressiveness.

### miR-4756 directly binds to circUBAP2 and the 3′-UTR of IFIT1/IFIT3

**Fig. 6:**
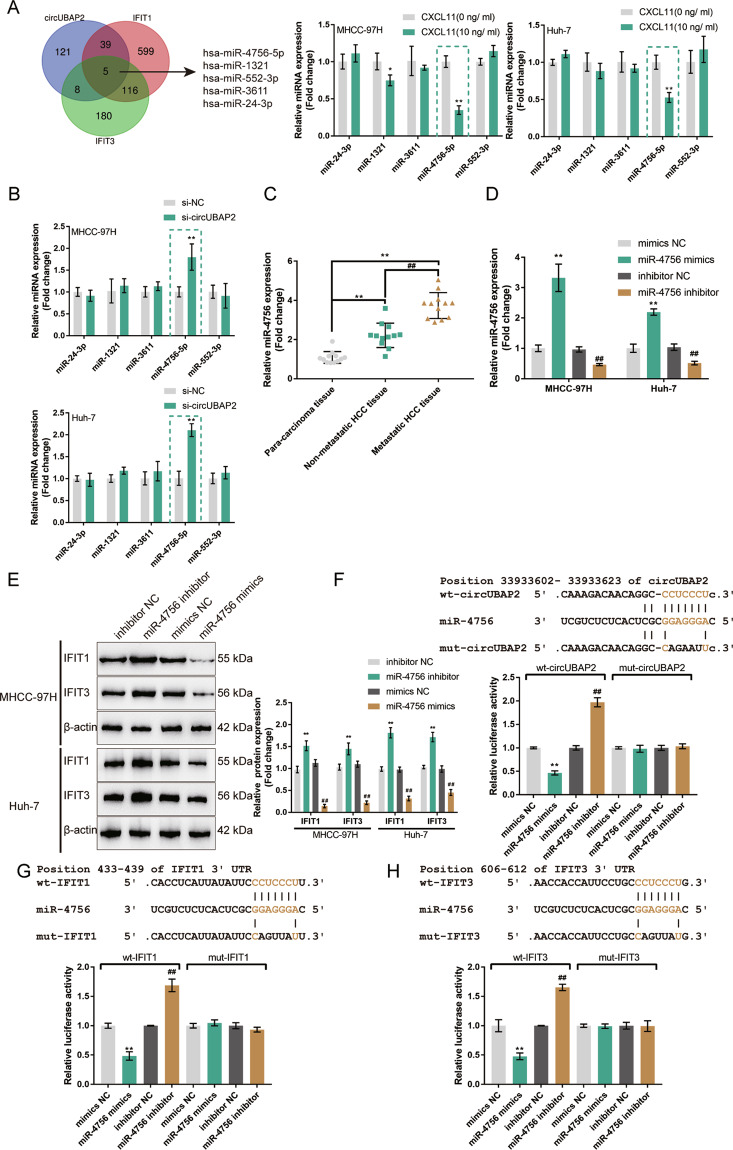
miR-4756 directly binds to circUBAP2 and the 3′-UTR of IFIT1/IFIT3. **A** ENCORI was used to predict miRNAs that circUBAP2 might target, and TargetScan was used to predict miRNAs that might target IFIT1/IFIT3. These two sets of miRNAs intersected at hsa-miR-4756-5p, hsa-miR-1321, hsa-miR-552-3p, hsa-miR-3611, and hsa-miR-24-3p. The expression of miR-4756-5p, miR-1321, miR-552-3p, miR-3611, and miR-24-3p was examined in MHCC-97H and Huh-7 cells with or without CXCL11 treatment. miR-4756 was chosen for further experiments due to its downregulation in both HCC cell lines in response to CXCL11 stimulation. **B** MHCC-97H and Huh-7 were transfected with si-circUBAP2 and examined for the expression of miR-4756 by real-time PCR. **C** The expression of miR-4756 was examined in 12 cases of nonmetastatic HCC tissues, metastatic HCC tissues, and para-carcinoma tissues by real-time PCR. **D** miR-4756 overexpression or inhibition was achieved in MHCC-97H and Huh-7 cells by transfecting miR-4756 mimics or miR-4756 inhibitor; the transfection efficiency was confirmed by real-time PCR. **E** MHCC-97H and Huh-7 cells were transfected with miR-4756 mimics or miR-4756 inhibitor and examined for the protein levels of IFIT1 and IFIT3 by Immunoblotting. **F**–**H** Wild- and mutant-type circUBAP2, IFIT1 3′-UTR, or IFIT3 3′-UTR luciferase reporter plasmids were constructed as described and named as wt-circUBAP2/mut-circUBAP2, wt-IFIT1/mut-IFIT1, and wt-IFIT3/mut-IFIT3. These plasmids were co-transfected in 293T cells with miR-4756 mimics or miR-4756 inhibitor and the luciferase activity was determined. *P < 0.05, **P < 0.01, compared to si-NC + inhibitor NC group. ^##^*P* < 0.01, compared to si-cirUBAP2+miR-4756 inhibitor group.

Here, we continued to search for miRNAs that might mediate the crosstalk between circUBAP2 and IFIT1/IFIT3 with ENCORI and TargetScan. These two predicted sets of miRNAs intersected at hsa-miR-4756-5p, hsa-miR-1321, hsa-miR-552-3p, hsa-miR-3611, and hsa-miR-24-3p (Fig. 6A). The expression of miR-4756-5p, miR-1321, miR-552-3p, miR-3611, and miR-24-3p was examined in MHCC-97H and Huh-7 cells with/without CXCL11 stimulation. As shown in Fig. 6A, only miR-4756 was significantly downregulated in both HCC cell lines upon CXCL11 stimulation. Moreover, only miR-4756 was dramatically increased in both HCC cell lines in the circUBAP2-silenced MHCC-97H and Huh-7 cells (Fig. 6B). And further experiments confirmed the expression of miR-4756 was significantly downregulated in the nonmetastatic HCC tissue samples and metastatic HCC tissue samples compared with the para-carcinoma tissue samples and increased in the metastatic HCC tissue samples than that in the nonmetastatic HCC tissue samples (Fig. 6C).

Next, miR-4756 overexpression or inhibition was achieved in MHCC-97H and Huh-7 cells by transfecting miR-4756 mimics or miR-4756 inhibitor, as confirmed by real-time PCR (Fig. 6D). In MHCC-97H and Huh-7 cells, miR-4756 overexpression decreased, while miR-4756 inhibition elevated the IFIT1 and IFIT3 protein levels (Fig. 6E). Thus, circUBAP2 negatively modulates miR-4756 expression, and miR-4756 negatively modulates IFIT1 and IFIT3 expression.

Then, we validated the binding of miR-4756 to circUBAP2 and IFIT1/IFIT3 with dual-luciferase reporter assay. We constructed wild-type and mutant-type circUBAP2 and IFIT1 3′-UTR and IFIT3 3′-UTR luciferase reporter plasmids as described and named them wt-circUBAP2/mut-circUBAP2, wt-IFIT1/mut-IFIT1, and wt-IFIT3/mut-IFIT3 (Fig. 6F–H). We co-transfected these plasmids into 293T cells with miR-4756 mimics/inhibitor, and examined luciferase activity. Figure 6F–H shows that miR-4756 overexpression dramatically decreased, while miR-4756 inhibition increased, the luciferase activity of wt-circUBAP2, wt-IFIT1, and wt-IFIT3; after we mutated the putative miR-4756 binding site, miR-4756 overexpression or miR-4756 inhibition failed to change the luciferase activity of mut-circUBAP2, mut-IFIT1, or mut-IFIT3 (Fig. 6F–H). Thus, miR-4756 is a direct target of circUBAP2 and IFIT1/IFIT3 are direct targets of miR-4756.

### Combined effects of circUBAP2 and miR-4756 on IFIT1/IFIT3 expression and the HCC cell phenotype

**Fig. 7:**
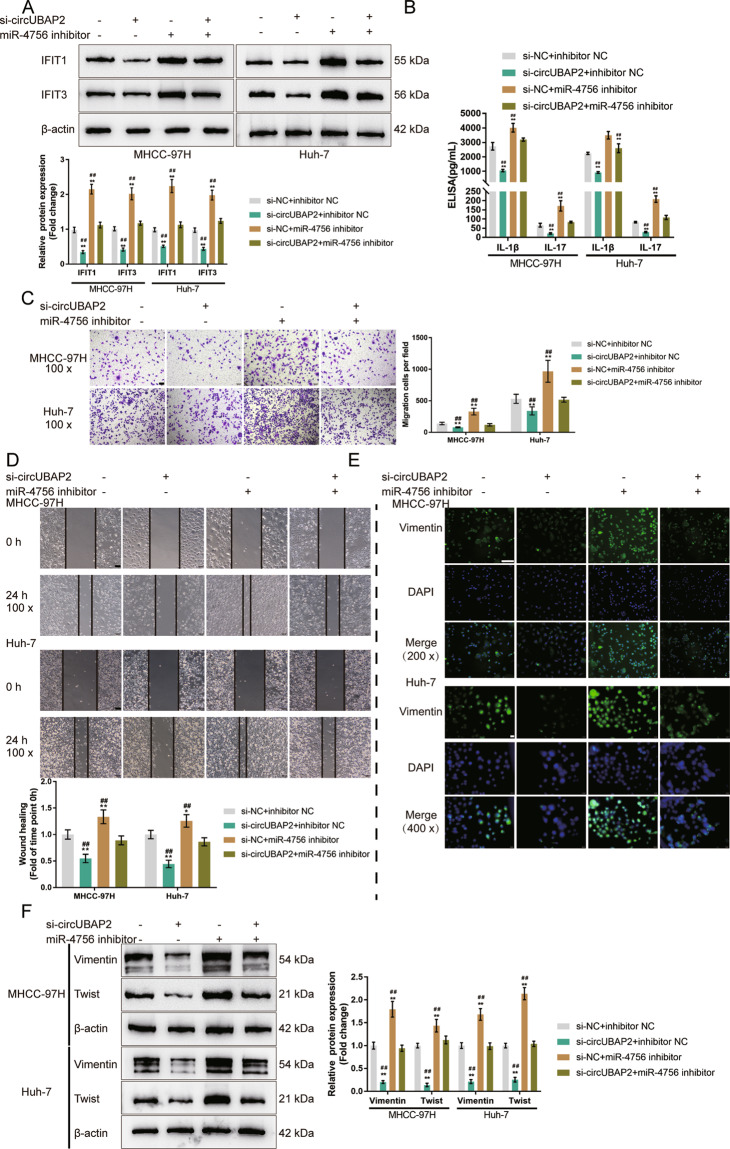
Combined effects—effects of circUBAP2 and miR-4756 on IFIT1/IFIT3 expression and HCC cell phenotype. MHCC-97H and Huh-7 cells were co-transfected with si-circUBAP2 and miR-4756 inhibitor and examined for **A** The protein levels of IFIT1 and IFIT3 by Immunoblotting; **B** The concentrations of IL-1β, and IL-17 in the culture medium by ELISA; **C** Cell migration by Transwell assay; **D** Cell migration by wound healing assay; **E** The cellular protein content and distribution of Vimentin was examined by IF staining; **F** The protein levels of Vimentin and Twist was examined by Immunoblotting. *P < 0.05, **P < 0.01, compared with the si-NC + inhibitor NC group; ^##^*P* < 0.01, compared with the si-circUBAP2 + miR-4756 inhibitor group.

To confirm whether circUBAP2, miR-4756, and IFIT1/IFIT3 could form a regulatory axis, we co-transfected MHCC-97H and Huh-7 cells with si-circUBAP2 and miR-4756 inhibitor and determined the IFIT1 and IFIT3 protein levels. As shown in Fig. 7A, circUBAP2 silencing significantly decreased, whereas miR-4756 inhibition increased, the IFIT1 and IFIT3 protein levels; miR-4756 inhibition significantly attenuated the effects of circUBAP2 knockdown. In summary, circUBAP2 acts as a ceRNA for miR-4756, competing with IFIT1/IFIT3 for miR-4756 binding and counteracting miR-4756-mediated IFIT1/IFIT3 inhibition.

Next, the coeffects of circUBAP2 and miR-4756 on HCC cell aggressiveness were examined. As shown in Fig. 7B, the medium concentrations of IL-1β and IL-17 were significantly reduced by circUBAP2 silencing but increased by miR-4756 inhibition; the effects of circUBAP2 silencing were partially reversed by miR-4756 inhibition. Transwell and wound healing assays indicated that circUBAP2 silencing inhibited HCC cell migration, whereas miR-4756 inhibition promoted HCC cell migration; miR-4756 inhibition significantly attenuated the effects of circUBAP2 silencing (Fig. 7C, D). Consistently, circUBAP2 silencing decreased, while miR-4756 inhibition elevated Vimentin and Twist levels; miR-4756 inhibition significantly attenuated the effects of circUBAP2 silencing (Fig. 7E, F). In summary, circUBAP2 participates in the functions of CAFs-derived CXCL11 through the circUBAP2/miR-4756/IFIT1/IFIT3 axis.

### Combined effects of circUBAP2 and miR-4756 on tumor growth and lung metastasis in the BALB/c mice with orthotopically implanted tumors

**Fig. 8:**
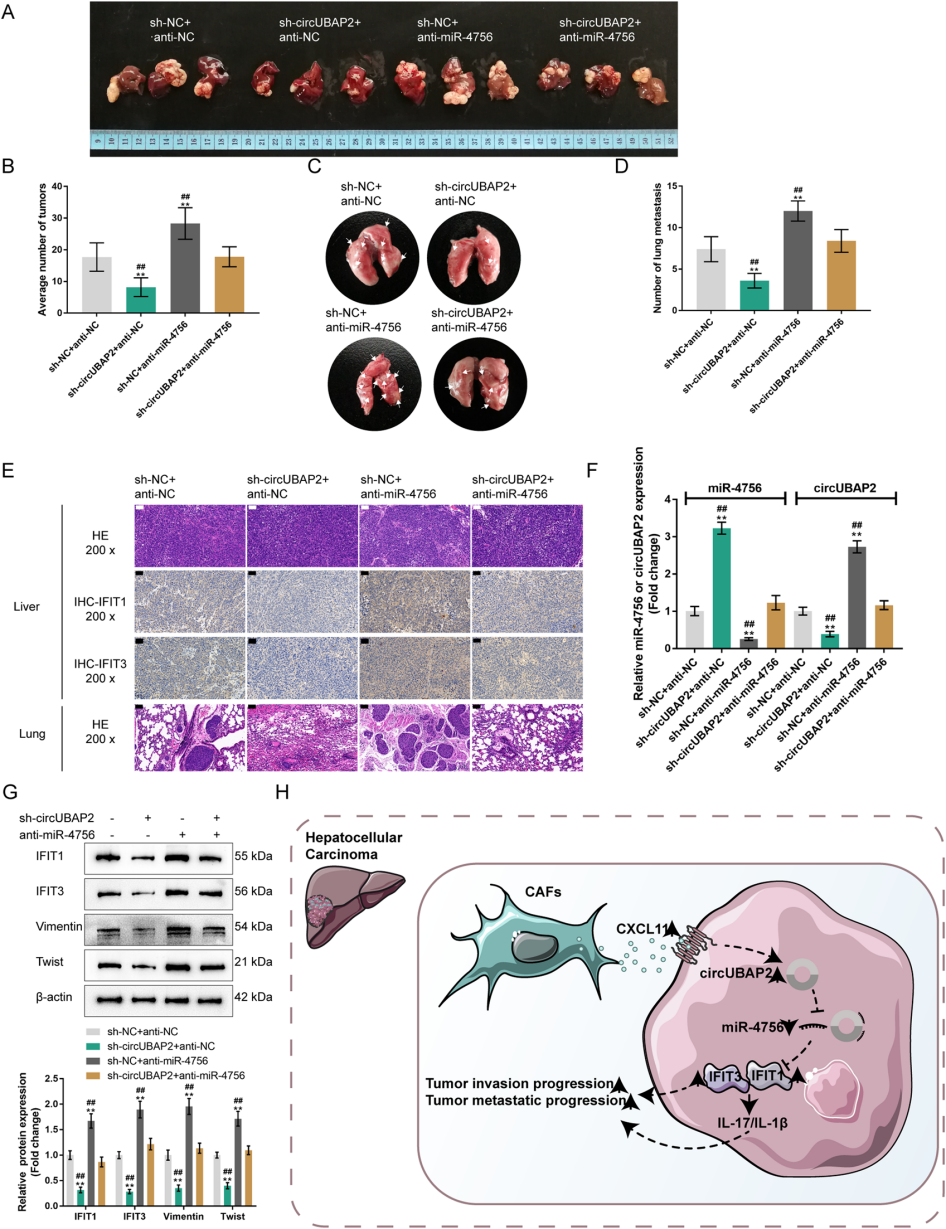
Combined effects of circUBAP2 and miR-4756 on tumor growth and lung metastasis in the orthotopically implanted tumor model in BALBc nude mice. **A**, **C** The orthotopic implantation tumor model was conducted by injecting Huh-7 cells coinfected with sh-NC/sh-circUBAP2 and anti-NC/anti-miR-4756 lentivirus into the left liver lobe of mice. After 5 weeks, anesthetized mice were sacrificed and livers and lungs were collected for further experiments. **B** Tumor numbers in mice livers from different groups were counted. **D** Lung metastatic nodules in mice from different groups were counted. **E** The histopathological characteristics of mice livers and lungs were evaluated by H&E staining. The protein content and distribution of IFIT1/3 in mice liver tumors were examined by IHC staining. **F** The expression levels of circUBAP2 and miR-4756 in mice livers from different groups were examined by qRT-PCR. **G** The protein levels of IFIT1/3, Vimentin, and Twist in mice livers tumors from different groups were examined by Immunoblotting. **H** A schematic graph of the mechanism. CXCL11 acts as a key mediator integrating CAFs and CAFs-cohabitating HCC cells, and also work as an extracellular remodeler promoting HCC migration and metastasis through the activation of circUBAP2/miR-4756/IFIT1/3 in tumor cells. **P < 0.01, compared with the sh-NC + anti-NC group; ^##^*P* < 0.01, compared with the sh-circUBAP2 + anti-miR-4756 group.

Finally, we established an orthotopic implantation tumor model by injecting Huh-7 cells coinfected with sh-NC/sh-circUBAP2 and anti-NC/anti-miR-4756 lentivirus into the left liver lobe of mice to investigate their effects on lung metastasis. Four weeks after the injection, anesthetized mice were sacrificed, and livers and lungs were collected for further experiments (Fig. 8A, C). As shown in Fig. 8B, the mice in the sh-circUBAP2 + anti-NC group developed the fewest liver tumor nodules, whereas the mice in the sh-NC + anti-miR-4756 group developed the most liver tumor nodules; the liver tumor nodules number in the mice from the sh-circUBAP2 + anti-miR-4756 group was almost the same as that in the sh-NC + anti-NC group. Similar results were observed for lung metastatic nodules (Fig. 8D), as confirmed by H&E staining, in which circUBAP2 silencing attenuated, whereas miR-4756 inhibition enhanced, the lung metastatic ability (Fig. 8E). Additionally, as shown by IHC staining and immunoblotting, the protein levels of IFIT1/3 were decreased by circUBAP2 knockdown but elevated by miR-4756 inhibition; miR-4756 inhibition significantly attenuated the effects of circUBAP2 knockdown on IFIT1/3 levels in the orthotopically implanted tumors (Fig. 8F, G). In mouse liver tumors, sh-circUBAP2 downregulated circUBAP2 expression and upregulated miR-4756 expression, whereas the miR-4756 inhibitor exerted the opposite effects; the effects of sh-circUBAP2 were partially reversed by miR-4756 inhibitor (Fig. 8F). Consistently, sh-circUBAP2 decreased, whereas the miR-4756 inhibitor elevated Vimentin and Twist in the mouse liver tumors; the effects of sh-circUBAP2 were partially reversed by the miR-4756 inhibitor (Fig. 8G).

## Discussion

CAFs overexpress proinflammatory genes and modulate the inflammatory microenvironment in the fibrotic liver, which could lead to tumor development and facilitate tumor progression^10,12^. Previously, Fang et al.^29^ demonstrated that activated CAFs further promote cancer progression by secreting proinflammatory cytokines, including IL-6 and IL-8. Herein, we detected dramatic increases in CXCL11 levels by CAFs when comparing the expression levels of different cytokines secreted by CAFs and NFs. CXCL11 is part of the superfamily of chemotactic cytokines and contributes to inflammatory responses, often through selective T cell recruitment to inflammatory sites. The results from other studies demonstrated a significantly elevated level of CXCL11 in HCC^30,31^ and indicated a role of CXCL11 in regulating the self-renewal and tumorigenicity of liver tumor-initiating cells^31^. Consistently, we also observed that in vitro cultivation with conditioned media of CAFs remarkably promoted the migratory capacity of HCC cells. And when additional CXCL11 was introduced into conditioned media, cancer cell migration was further enhanced. These in vitro findings were then confirmed by in vivo investigations that CAFs increased the number of tumor metastases in the mice bearing Huh-7 cell-derived tumors; when CXCL11 was silenced, the effects of CAFs on tumor progression were significantly attenuated. The present findings were consistent with previous findings by Liu et al.^12^ that CAFs promote the migration and invasion of HCC cells in vitro and facilitate HCC metastasis to the bone, brain and lung in NOD/SCID mice. These results fundamentally confirmed a key role of CXCL11 in mediating the crosstalk between CAFs and HCC progression.

Increasing evidence demonstrates the possible involvement of noncoding RNAs in the actions of cytokines secreted by CAFs. However, previous studies mostly focused on the mechanisms underlying tumor progression by long noncoding RNAs and miRNAs^19^, while less is known about the functions and mechanisms of CAF-related circRNAs in HCC. Herein, we performed RNA sequencing and analyzed the differentially expressed circRNAs and mRNAs between CXCL11-stimulated and unstimulated HCC cells. Of these differentially expressed circRNAs, circUBAP2 showed a much greater potential to facilitate HCC progression than other circRNAs according to its expression status in HCC and its roles in previous studies of other diseases from the literature. The oncogenic role of circUBAP2 has already been reported and found to work as a ceRNA. circUBAP2 modulates pancreatic adenocarcinoma tumorigenesis by regulating the levels of hsa-miR-494 and the expression of CXCR4, HIF1A, ZEB1, SDC1, and TWIST1^32^. In addition, circUBAP2 modulates cancer cell malignancy through the miR-641/YAP1 axis and miR-361-3p/SOX4 axis in osteosarcoma and cervical cancer, respectively^33,34^. Consistently, our results showed that circUBAP2 silencing was sufficient to attenuate HCC cell migration, as well as the promotive effects of CXCL11 on HCC cell migration.

Intriguingly, our RNA sequencing also identified a number of mRNAs deregulated in the CXCL11-stimulated HCC cells, and the signaling and function annotation results suggested that upregulated mRNAs were dramatically enriched in IL-17 signaling and the cell response to interferon. These annotations were consistent with the fact that CXCL11 belongs to the class of IFN-γ inducible chemokines, which are notably elevated in the process of hepatic inflammation^35^. Likewise, Helbig et al.^36^ revealed that stimulation of Huh-7 cells with IFN-γ could also produce high levels of CXCL11 in vitro, indicating that IFN-γ might serve as a key regulatory factor of CXCL11 in malignant and inflammatory hepatocytes. Given these findings, we next focused on the interferon signaling related gene IFIT1/3^37^ for further investigations. si-circUBAP2 downregulated IFIT1/3 expression, further confirming the role of circUBAP2 in regulating the expression of IFIT1/3 as a ceRNA. Indeed, silencing either IFIT1 or IFIT3 reduced the IL-17 and IL-1β levels and attenuated the aggressiveness of HCC cells.

We then intended to identify miRNAs that might form a ceRNA regulatory network with circUBAP2 and IFIT1/3, and with predictive tools, we succeeded in identifying miR-4756 as a potential participant. By competing with IFIT1/3 for miR-4756 binding, circUBAP2 attenuated miR-4756-induced IFIT1/3 inhibition. Despite a previous report that exosomal miR-320a^38^ and miR-21^39^ from CAFs mediate the crosstalk between CAFs and HCC cells, we are the first to demonstrate that CXCL11 stimulation induces the deregulation of miR-4756 in HCC cells, which might subsequently be involved in the role of CXCL11 in HCC cell migration. In contrast to circUBAP2 and IFIT1/3, miR-4756 expression was significantly downregulated in HCC cells in response to CXCL11 stimulation. Regarding cellular functions, miR-4756 inhibition promoted HCC cell migration, and the effects of circUBAP2 silencing were partially reversed by miR-4756 inhibition. miR-4756 inhibition also reversed the effects of circUBAP2 silencing on metastatic progression in mice bearing Huh-7 cell-derived tumors.

In summary, our work suggests a mechanism whereby CXCL11 acts as a key mediator integrating CAFs and CAF-cohabitating cancer cells, and functions as an extracellular remodeler promoting HCC migration and metastasis through the activation of circUBAP2/miR-4756/IFIT1/3 in tumor cells (Fig. 8H).

## Materials and methods

### Clinical sampling

Twelve HCC samples with metastasis (metastatic HCC; including portal vein invasion, extrahepatic distant metastasis, capsule invasion, vascular tumor thrombus, portal vein tumor thrombus, lymph node metastasis) and 12 HCC samples without metastasis (nonmetastatic HCC) were collected. Postoperative pathology confirmed the diagnosis of HCC, and none of the patients received preoperative radiotherapy or TACE (transarterial chemoembolization). In addition, for 12 cases, adjacent hepatic tissues were collected from these HCC patients with metastases as negative controls. All samples were collected from patients who received surgical resection at Zhongshan Hospital, Fudan University. Informed consent was signed and obtained from all patients enrolled. All clinical sampling was performed with the approval of the Ethics Committee of Zhongshan Hospital, Fudan University. Tissues were stored at −80 °C immediately after harvesting until further processing.

### CAF and NF isolation from HCC samples and identification

Five fresh HCC samples and their corresponding nontumor controls were washed with serum-free DMEM/F-12 medium, cut into 0.2 × 0.2 mm fragments, and incubated in a fresh culture medium for 24 h for attachment. After incubation, the unattached cells were removed and the remaining cells were allowed to grow on the plate for 2–3 weeks. During this period, the medium was replenished every 2 days until fibroblasts began to grow out. To ensure that there was no contamination of other cell types, we verified that the fibroblasts for >90% positive for the fibroblast marker α-SMA and negative for endothelial, HCC, and epithelial cell markers, including CD31, AFP and pan-cytokeratin, respectively. Fibroblasts isolated from tumor tissues were defined as CAFs, and those isolated from nontumor counterparts were defined as NFs. CAFs and NFs of less than ten generations were used for experiments.

### Preparation of conditioned medium (CM)

CAFs or NFs were seeded on six-well plates at a density of 1 × 10^5^ cells/well. After 24 h of cell seeding, the medium was removed, the cells were washed once with PBS, and 1 ml of serum-free medium was added to each well. Then, the cells were incubated at 37 °C for 24 h, the CM was collected, and the collected CM was passed through a 0.2 μm membrane syringe filter to remove all cells and cell debris. Then, the CM was collected for further use.

### Immunofluorescence (IF) staining

HCC cells and collected CAFs and NFs were fixed with 4% paraformaldehyde for 20 min, washed with PBS and permeabilized with 0.1% Triton X-100 for 10 min. Then, the cells were blocked in PBS containing 10% BSA at room temperature for 1 h. At the end of the blocking, the CAFs and NFs were incubated with anti-α-SMA (55135-1-AP; Proteintech, Wuhan, China) or anti-Vimentin (10366-1-AP, Proteintech) at 4 °C overnight. HCC cells were incubated with anti-Vimentin (10366-1-AP, Proteintech) at 4 °C overnight. Then, the cells were stained with an appropriate FITC-conjugated secondary antibody (1:1000) in a dark room at 37 °C for 1 h. Then, DAPI was used to stain the nucleus, and the cover glass was sealed with Antifade Mounting Medium.

### HCC cell line

A metastatic HCC cell line, MHCC-97H, was established and provided by the Liver Cancer Institute, Fudan University (Shanghai, China)^40^ and cultured in Dulbecco’s modified Eagle’s medium (DMEM) containing 4 mM L-glutamine and 1 g/l glucose and supplemented with 10% FBS (Cat. # 10099, GIBCO, Carlsbad, CA, USA). A metastatic HCC cell line, Huh-7 (JCRB0403), was obtained from the Japanese Cancer Research Resources Bank (JCRB; Osaka, Japan) and cultured in DMEM containing 4 mM L-glutamine and 1 g/L glucose and supplemented with 10% FBS (Cat. # 10099, Gibco).

MHCC-97H cells were cultured in different conditioned media for 72 h and evaluated at different magnifications using scanning electron microscopy (SEM; Regulus 8100, Hitachi, Tokyo, Japan).

### Cell treatment

MHCC-97H and Huh-7 cells were cultured with NF-CM, CAF-CM or CAF-CM containing different concentrations of CXCL11 recombinant protein (10876-HNAE, SinoBiological, China) or CXCL11 neutralizing antibody (MAB672, R&D Systems, USA) for 72 h. Then, Transwell, wound healing, IF, and western blot assays were performed.

### RNA sequencing and bioinformatics analysis

MHCC-97H cells were treated with or without CXCL11 (10 ng/ml) for 72 h. Then, the total RNA was isolated by TRIzol (Invitrogen). Next-generation sequencing was performed by Aksomics, Inc. (Shanghai, China) using an Illumina HiSeq 4000 system (USA). The differentially expressed genes (mRNAs and circRNAs) analysis was performed using DESeq R package. The genes with threshold values of |log2FC| > 0.56 and a Benjamini–Hochberg corrected *P* value of 0.05 were selected. Finally, these differentially expressed genes were subjected to Gene Ontology (GO) analysis (http://geneontology.org/) and Kyoto Encyclopedia of Genes and Genomes (KEGG) signaling annotation (https://www.genome.jp/kegg/).

### Cell transfection

For CXCL11 knockdown in CAFs, the sh-CXCL11 vector was obtained from GenePharma (Shanghai, China), and the sh-NC vector was used as the negative control. For miR-4756 overexpression or inhibition in HCC cells, miR-4756 mimics or inhibitor was synthesized and obtained from GenePharma; mimics NC or inhibitor NC was used as a negative control. For circUBAP2, IFIT1, or IFIT3 silencing, small interfering RNA targeting circUBAP2 (si1/2-circUBAP2; si-NC as a negative control), IFIT1 (si1/2-IFIT1; si-NC as a negative control), or IFIT3 (si1/2-IFIT3; si-NC as a negative control) was synthesized and obtained from GenePharma. Then, these vectors, siRNAs, miRNA mimics, or inhibitor were transfected into target cells with Lipofectamine 3000 Reagent (Thermo Fisher Scientific, Waltham, MA, USA) following the manufacturer’s protocol. The siRNA or miRNA sequences are presented in Table S1.

### Real-time quantitative polymerase chain reaction (real-time qPCR)

Cells were digested and lysed, and total RNA was extracted according to the RNA isolation kit instructions (Toyobo, Tokyo, Japan). A total of 5 μl of RNA was obtained from each group, and ultrapure water of RNase was added to dilute the substance. Reverse transcription was performed according to the Fermentas RevertAid First Strand cDNA Synthesis kit instructions (Thermo Scientific). Amplification reactions were performed using the Universal SYBR Green Master system (Roche, Basel, Switzerland). The expression levels were calculated using the 2^−ΔΔCt^ method. All the primers used in qRT-PCR are shown in Table S1.

### Immunoblotting

After 72 h of transfection, cells were added with an appropriate amount of RIPA (PMSF) buffer. Then, the total protein was extracted by centrifugation, the total protein concentration was determined using the BCA method, and the protein was separated using SDS-PAGE. The proteins were electrotransferred to PVDF membranes and blocked with 5% milk blocking solution for 1 h to block nonspecific bindings. The membranes were incubated with the following primary antibodies overnight at 4 °C and washed three times with 0.05% TBST for 5 min each time: CXCL11 (CSB-PA06119A0Rb; Cusabio, Wuhan, China), Vimentin (10366-1-AP; Proteintech, Wuhan, China), Twist (CSB-PA025358LA01HU, Cusabio), β-actin (60008-1-Ig, Proteintech), IFIT1 (CSB-PA011018LA01HU, Cusabio), and IFIT3 (CSB-PA011022HA01HU, Cusabio). The membranes were then incubated with secondary antibodies (HRP-labeled goat anti-rabbit IgG and goat anti-mouse IgG) at room temperature for 1 h and washed three times with 0.05% TBST for 5 min each time. The signal was detected by the ECL chemiluminescence method.

### H&E staining

Tissue samples were fixed in 4% paraformaldehyde, embedded in paraffin, and cut into 4-μm thick sections. H&E staining was performed to observe the histopathological features^41^. At least five fields were analyzed on each section, and an Olympus microscope captured images.

### Immunohistochemical (IHC) staining

After dewaxing and hydration, the tissue sections were permeabilized with 0.2% triton (Sigma, St. Louis, MO, USA) at room temperature for 10 min, and then incubated with blocking solution (3.75% BSA/5% goat serum; Zymed, Carlsbad, CA, USA) for 30 min. Next, samples were incubated with anti-CXCL11 (CSB-PA06119A0Rb, Cusabio). All sections were incubated with poly-HRP antibody for 30 min. Then, sections were stained by DAB staining kit (Boster, Wuhan, China), and then stained with hematoxylin (0.2 mg/ml, Sigma) to label the nucleus. Finally, sections were observed under a microscope.

### MTT assay

HCC cells (5 × 10^3^ cells per well in 96-well plates) were added to each well of the 96-well plates. At each time point, 20 μl MTT (5 mg/ml; Sigma-Aldrich, St. Louis, MO, USA) was then added to each well and followed by incubation for another 4 h in a humidified incubator. After removing the supernatant, DMSO was used to dissolve the formazan. The OD values were determined at 450 nm.

### EdU assay

Cells were cultured in different condition medium containing EdU solution (final concentration of 50 μM, RioboBio, Shanghai, China) for 2 h. At the end of the incubation, cells were collected and fixed with 1 ml 4% paraformaldehyde. After washing with PBS, each well was added with 1 ml 0.5% TritonX-100 and incubated at room temperature for 10 min. After washing with PBS, each well was added with 1× Apollo^®^ staining agent (RioboBio) and incubated at room temperature for 10 min in the dark. Then, cells were collected and resuspended with 500 μl PBS and analyzed by flowcytometry.

### Transwell cell migration assays

The migration analysis was performed using Transwell plates without Matrigel gel in the top chambers. Target cells were transfected and digested with 0.25% trypsin, suspended in serum-free medium and counted. Then, 200 μl (5 × 10^5^ cells/ml) of transfected cells were plated in the upper chamber, and 600 µl of DMEM containing 10% FBS was added to the lower chamber. After 24 h of incubation, the upper chamber was washed twice with PBS. The cells on the upper surface were discarded with a cotton swab and the cells left were fixed with anhydrous methanol for 30 min and stained with 0.1% crystal violet for 30 min. The number of cells passing through the basement membrane was counted under an optical microscope and the representative images are shown.

### Cell migration by wound healing assays

Approximately 5 × 10^4^ target cells were plated in six-well plates. Then, a pipette tip was used to draw a gap on the cell monolayers. Mitomycin C was added to inhibit cell proliferation and, after 24 h, cells that migrated into the cleared section were observed under a microscope at specific time points.

### Animal ethics

All animal experiments complied with the Guidelines for the Care and Use of Experimental Animals and were approved by the Experimental Committee of Zhongshan Hospital, Fudan University.

### Animals

BALB/c nude male mice were obtained from SJA Laboratory Animal Co., Ltd (Shanghai, China). Mice were kept separately in plastic cages at 22–25 °C in a relative humidity of 50–70%. Mice were housed under a constant light–dark cycle and were allowed free access to food and water.

### Establishment of an orthotopic implantation model of HCC in mice

An orthotopic implantation model of HCC in BALB/c mice was established according to a previous report^42^. In general, CAFs were transfected with sh-NC or sh-CXCL11 vector for 72 h. Subsequently, mice were randomly divided into five groups: the Huh-7 cell group (*n* = 6), the Huh-7 cells + NFs group (*n* = 6), the Huh-7 cells CAFs (untransfected; *n* = 6) group, the Huh-7 cells + CAFs (transfected with sh-NC; *n* = 6) group, and the Huh-7 cells + CAFs (transfected with sh-CXCL11; *n* = 6) group. A total of 5 × 10^5^ Huh-7 cells alone or mixed with 5 × 10^5^ NFs or 5 × 10^5^ transfected/untransfected CAFs were suspended in 100 μL PBS and injected into nude mouse liver. After 2 weeks, anesthetized mice were sacrificed and the tumor volume and weight were examined. Tumor tissues were collected and subjected to H&E staining for the examination of the histopathological characteristics and IHC staining for CXCL11 levels in each group.

For determination of the combined effects of circUBAP2 and miR-4756 on HCC lung metastasis, 2 × 10^6^ Huh-7 cells were coinfected with sh-NC/sh-circUBAP2 and anti-NC/anti-miR-4756 lentivirus (GeneChem, China) and injected orthotopically into the left liver lobe of mice. After 5 weeks, anesthetized mice were sacrificed, in vivo tumor growth and lung metastases were detected, and the levels of related factors were examined.

### ELISA

The culture medium supernatant from NFs or CAFs was collected and centrifuged (1500 rpm for 5 min). Subsequently, the secretion of CXCL11 was detected using the ELISA kit (Invitrogen, USA). HCC cells were transfected with siRNAs or miR-4756 inhibitors for 72 h. Then, the supernatant was collected and centrifuged (1500 rpm for 5 min). Subsequently, the secretion of IL-17 and IL-1β into the conditioned medium was detected using ELISA kits following the manufacturer’s instructions (R&D Systems, Minneapolis, MN, USA).

### Dual-luciferase reporter assay

IFIT1/IFIT3 3′-UTR or circUBAP2 sequence containing the miR-4756 binding sites were fused into psiCHECK-2 luciferase reporter vector (Promega) to generate wild-type reporter vector (wt-IFIT1/IFIT3 3′-UTR or wt-circUBAP2); moreover, site-directed mutagenesis of the miR-4756 binding sites in the IFIT1/IFIT3 3′-UTR or circUBAP2 sequence was introduced into the luciferase reporter vector to generate the mutant-type reporter vector, (mut-IFIT1/IFIT3 3′-UTR or mut-circUBAP2). Then, 293T cells were co-transfected with miR-4756 mimics/inhibitor together with these reporter vectors using Lipofectamine 3000. The luciferase activity was determined 24 h post transfection using the Dual-Luciferase Reporter System (Promega) as directed by the manufacturer’s instructions.

### Statistics analysis

Results are representative of at least three independent experiments with no less than triplicates for each group unless specified. Two public datasets, GSE14323 and GSE6764, were downloaded from the Gene Expression Omnibus and further analyzed. Data were processed with GraphPad (San Diego, California, USA) and expressed as mean ± standard deviation (S.D.). A one-way analysis of variance (ANOVA) and Tukey’s multiple comparison test, or Student’s *t*-test, were used to assess its statistical significance. *P* value < 0.05 is considered statistically significant.

## Supplementary information

Supplemental Figure S1

Supplemental Figure S2

fig.S3

fig.S4

Supplemental Table S1

Supplemental Table S2

supplementary figure and table legends

## Data Availability

Please contact the authors for data requests.
