# Supplementary figures and images for: Cancer-associated fibroblast-derived CXCL11 modulates hepatocellular carcinoma cell migration and tumor metastasis through the circUBAP2/miR-4756/IFIT1/3 axis
Source: Cell Death Dis. 2021 Mar 11;12(3):260. doi: 10.1038/s41419-021-03545-7 (PMC7952559; doi:10.1038/s41419-021-03545-7)

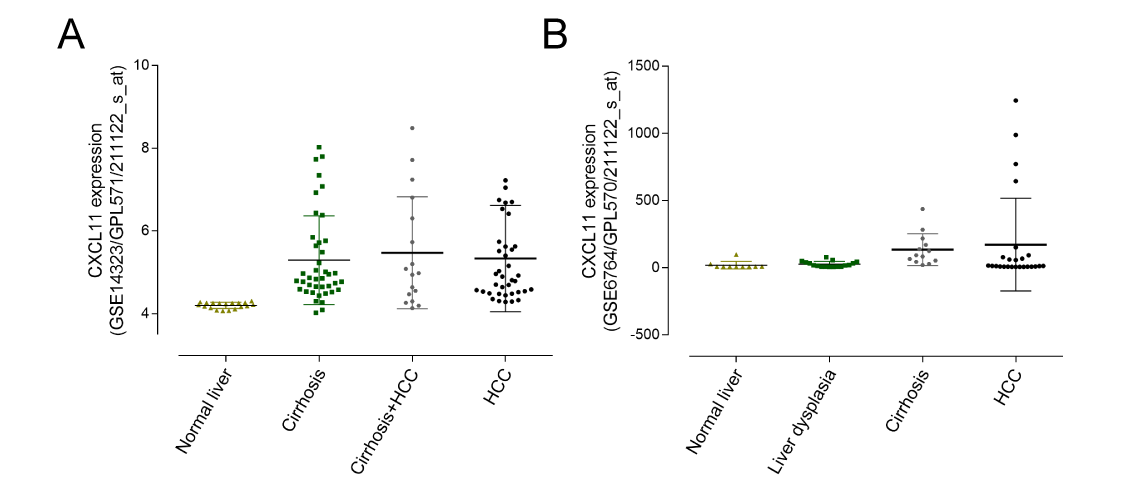

Supplement: Supplementary file 1 — Supplemental Figure S1 [file 41419_2021_3545_MOESM1_ESM.tif]

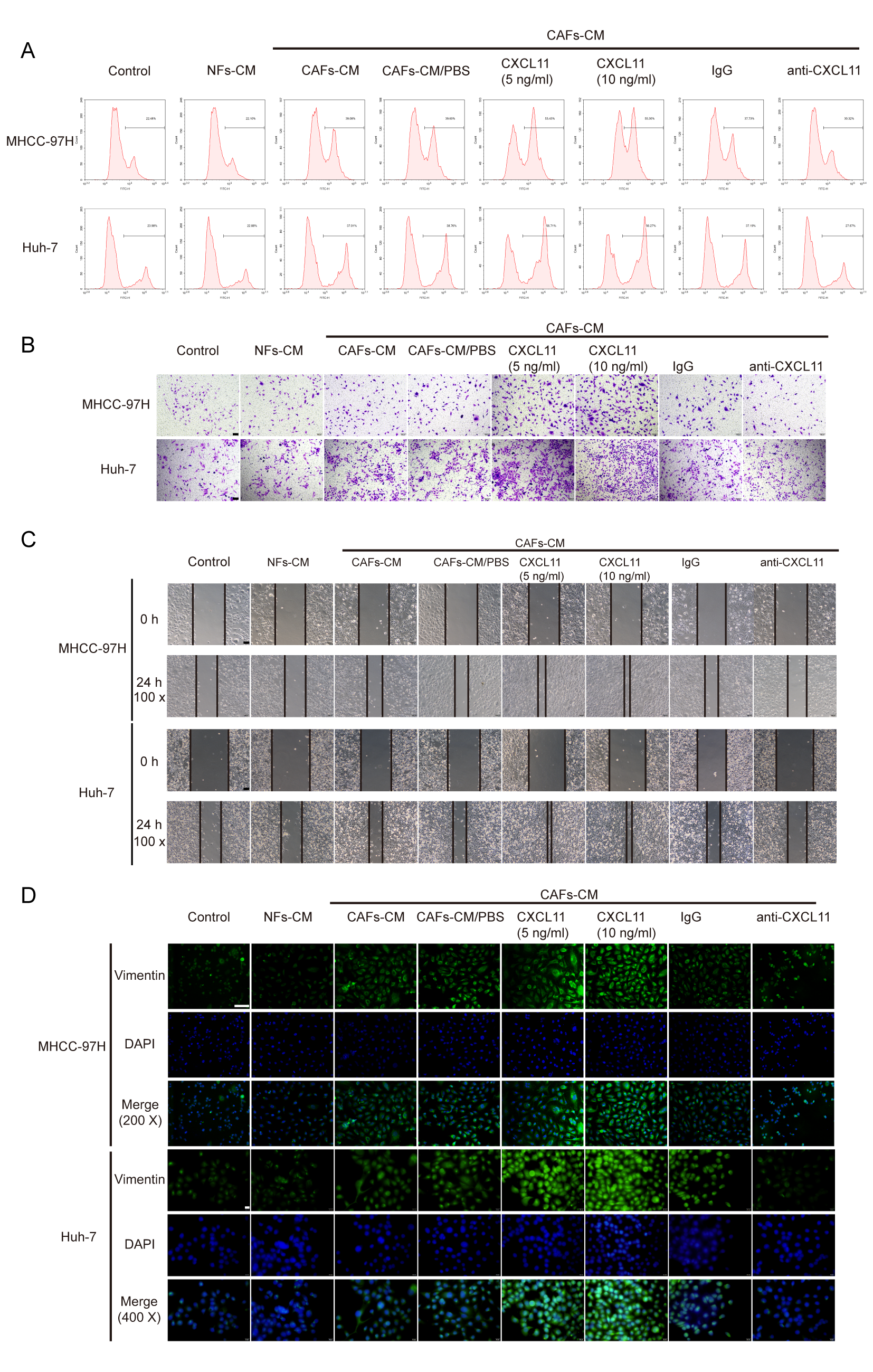

Supplement: Supplementary file 2 — Supplemental Figure S2 [file 41419_2021_3545_MOESM2_ESM.tif]

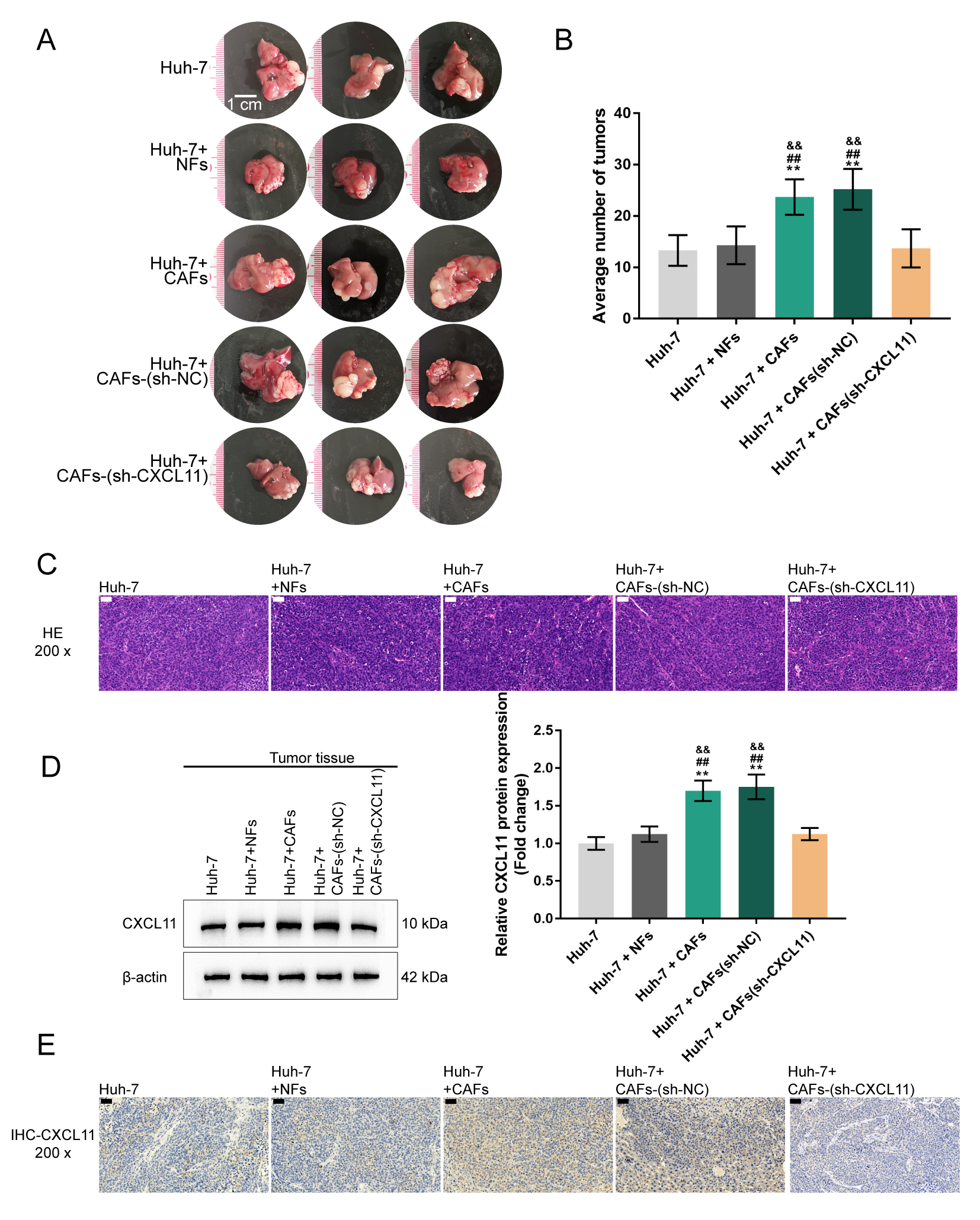

Supplement: Supplementary file 3 — fig.S3 [file 41419_2021_3545_MOESM3_ESM.tif]

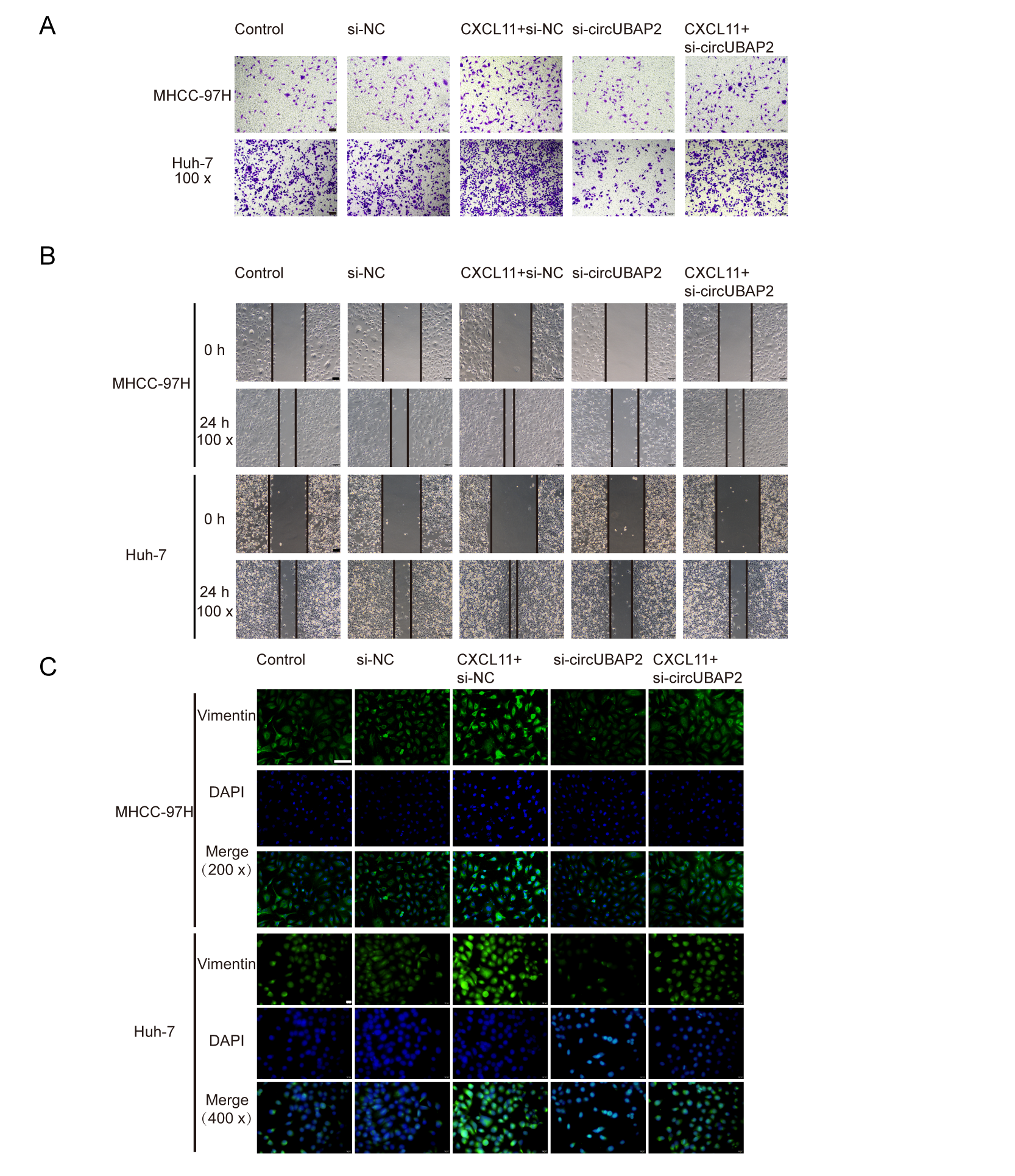

Supplement: Supplementary file 4 — fig.S4 [file 41419_2021_3545_MOESM4_ESM.tif]
