# Supplementary material for: Cancer-associated fibroblast-derived CXCL11 modulates hepatocellular carcinoma cell migration and tumor metastasis through the circUBAP2/miR-4756/IFIT1/3 axis
Source: Cell Death Dis. 2021 Mar 11;12(3):260. doi: 10.1038/s41419-021-03545-7 (PMC7952559; doi:10.1038/s41419-021-03545-7)
Supplement: Supplementary file 5 — Supplemental Table S1 [file 41419_2021_3545_MOESM5_ESM.docx]

Table S1 Primers sequence

| Name | forward | reverse |
| --- | --- | --- |
| RT-PCR  CCL2 | CAGCCAGATGCAATCAATGCC | TGGAATCCTGAACCCACTTCT |
| RT-PCR  CCL5 | CCAGCAGTCGTCTTTGTCAC | CTCTGGGTTGGCACACACTT |
| RT-PCR  CCL7 | ATACTTCAACTACCTGCTGC | GGCTACTGGTGGTCCTTCT |
| RT-PCR  CCL8 | TGGAGAGCTACACAAGAATCACC | TGGTCCAGATGCTTCATGGAA |
| RT-PCR  CCL11 | GACGCTGTCTTTGCATAGGC | GGATTTAGGCATCGTTGTCCTTT |
| RT-PCR  CCL12 | ATTCTCAACACTCCAAACTGTGC | ACTTTAGCTTCGGGTCAATGC |
| RT-PCR  CCL13 | GCTTGAGGTGTAGATGTGTCC | CCCACGGGGCAAGATTTGAA |
| RT-PCR  CCL14 | CGCTACAGCGACGTGAAGAA | GTTCCAGGCGTTGTACCAC |
| RT-PCR  CXCR3 | CCACCTAGCTGTAGCAGACAC | AGGGCTCCTGCGTAGAAGTT |
| RT-PCR  IFIT1 | TTGATGACGATGAAATGCCTGA | CAGGTCACCAGACTCCTCAC |
| RT-PCR  IFIT3 | TCAGAAGTCTAGTCACTTGGGG | ACACCTTCGCCCTTTCATTTC |
| RT-PCR  MiR-24-3p | RT:GTCGTATCCAGTGCGTGTCGTGGAGTCGGCAATTGCACTGGATACGACCTGTTC  F: GCTGGCTCAGTTCAGCAG | CAGTGCGTGTCGTGGA |
| RT-PCR  MiR-1321 | GTCGTATCCAGTGCGTGTCGTGGAGTCGGCAATTGCACTGGATACGACATCACA  GCGCAGGGAGGTGAA | CAGTGCGTGTCGTGGA |
| RT-PCR  MiR-3611 | GTCGTATCCAGTGCGTGTCGTGGAGTCGGCAATTGCACTGGATACGACTAAGAA  GCCGTTGTGAAGAAAGAAA | CAGTGCGTGTCGTGGA |
| RT-PCR  MiR-4756-5p | GTCGTATCCAGTGCGTGTCGTGGAGTCGGCAATTGCACTGGATACGACAGCAGA  CAGGGAGGCGCTCACTC | CAGTGCGTGTCGTGGA |
| RT-PCR  MiR-552-3p | GTCGTATCCAGTGCGTGTCGTGGAGTCGGCAATTGCACTGGATACGACTTGTCT  GCCAACAGGTGACTGGTT | CAGTGCGTGTCGTGGA |
| RT-PCR  U6 | CTCGCTTCGGCAGCACA | AACGCTTCACGAATTTGCGT |
| RT-PCR  CircUBAP2 | TTTCTCTTGCCCAATCCCCACC | CCAGATCAATGCTTTGCCCAGG |
| RT-PCR  CircRERE | AAGACGAGGACAATGACAACAATA | CTTCTTGCGATGGATGAACTATTT |
| RT-PCR  CircPPP6R3 | AAATGAGTCAGCCATAGTCAGTGC | CTTCTTGCGATGGATGAACTATTT |
| Si-NC | UUCUCCGAACGUGUCACGUTT | ACGUGACACGUUCGGAGAATT |
| Si-CircUBAP2 1# | AGAAAAUCUGCCAAUGUGCTT | GCACAUUGGCAGAUUUUCUTT |
| Si-CircUBAP2  2# | UGAAAUCUGUGGUUUUUCCTT | GGAAAAACCACAGAUUUCATT |
| Wt-circUBAP2 vector construction primer | aattctaggcgatcgctcgagATGACCAGAGTTCTGTGCATAACAG | attttattgcggccagcggccgcCTGCATGTGCAGAGAGGCTGC |
| mut-circUBAP2 vector construction primer | AACAGGCcgagggtCTGCCCTCCCGTCTGTGAG | CAGaccctcgGCCTGTTGTCTTTGGAGTGTCTAG |
| Wt-IFIT1 3’UTR vector construction primer | aattctaggcgatcgctcgagATTGTGAAATAAAAATAAAATCCTTAGCT | attttattgcggccagcggccgcCAGTTGTCATGTGGAAACCGC |
| mut-IFIT1 3’UTR vector construction primer | CcagttatTGCTAACTGCCATTGGACTTTTTC | CAGTTAGCAataactgGAATATAATGAGGTGTGAATGATCTCTCA |
| Wt-IFIT3 3’UTR vector construction primer | aattctaggcgatcgctcgagAGAGTTGTTTTCTCATGTTCATTATAGTTC | attttattgcggccagcggccgcTACATGCCTGAAGCTATAAGTGAGATAA |
| mut-IFIT3 3’UTR vector construction primer | CCTGCcagttatGCCAAGGGTCATAAATGGTGAC | TTGGCataactgGCAGGAATGGTGGTTATATTGTGA |
| Inhibitor NC | CAGUACUUUUGUGUAGUACAA |  |
| MiR-4756-5p inhibitor | AGCAGAGAGUGAGCGCCUCCCUG |  |
| Mimics NC | UUCUCCGAACGUGUCACGUTT | ACGUGACACGUUCGGAGAATT |
| MiR-4756-5p mimics | CAGGGAGGCGCUCACUCUCUGCU | CAGAGAGUGAGCGCCUCCCUGUU |
| Lv-sh-circUBAP2 lentivirus | GATCCGCAGATGTCACAGGATTAAATCTCGAGATTTAATCCTGTGACATCTGCTTTTTG | AATTCAAAAAGCAGATGTCACAGGATTAAATCTCGAGATTTAATCCTGTGACATCTGCG |
| Lv-sh-NC lentivirus | GATCCGCCAAGCCCTTGTCTTCACAACTCGAGTTGTGAAGACAAGGGCTTGGCTTTTTG | AATTCAAAAAGCCAAGCCCTTGTCTTCACAACTCGAGTTGTGAAGACAAGGGCTTGGCG |
| Lv-anti-miR-4756-5p | AGCAGAGAGTGAGTCCTCCCTGCGCGAGCAGAGAGTGAGTCCTCCCTGCGCGAGCAGAGAGTGAGTCCTCCCTGCGCGAGCAGAGAGTGAGTCCTCCCTGCGCGAGCAGAGAGTGAGTCCTCCCTGCGCGAGCAGAGAGTGAGTCCTCCCTGCGCGAGCAGAGAGTGAGTCCTCCCTGCGCGAGCAGAGAGTGAGTCCTCCCTGCGCG |  |
| Lv-anti-miR-NC | CAGTACTTTGTGTAGTACAACGCGCAGTACTTTGTGTAGTACAACGCGCAGTACTTTGTGTAGTACAACGCGCAGTACTTTGTGTAGTACAACGCGCAGTACTTTGTGTAGTACAACGCGCAGTACTTTGTGTAGTACAACGCGCAGTACTTTGTGTAGTACAACGCGCAGTACTTTGTGTAGTACAACGCG |  |
| sh-CXCL11 | GATCCGCTGTGATATTGTGTGCTACACTCGAGTGTAGCACACAATATCACAGCTTTTTG | AATTCAAAAAGCTGTGATATTGTGTGCTACACTCGAGTGTAGCACACAATATCACAGCG |
| sh-NC | GATCCAGCAAAGCTGAAGTAGCAGCACTCGAGTGCTGCTACTTCAGCTTTGCTTTTTTG | AATTCAAAAAAGCAAAGCTGAAGTAGCAGCACTCGAGTGCTGCTACTTCAGCTTTGCTG |
