# Supplementary material for: Cancer-associated fibroblast-derived CXCL11 modulates hepatocellular carcinoma cell migration and tumor metastasis through the circUBAP2/miR-4756/IFIT1/3 axis
Source: Cell Death Dis. 2021 Mar 11;12(3):260. doi: 10.1038/s41419-021-03545-7 (PMC7952559; doi:10.1038/s41419-021-03545-7)
Supplement: Supplementary file 6 — Supplemental Table S2 [file 41419_2021_3545_MOESM6_ESM.docx]

**Table S2 Up-regulation of KEGG pathway table**

| ID | Term | WebSite | Count | Size | numInt | numTotal | p_value | fdr | Enrichment_Score | GeneRatio | Genes |
| --- | --- | --- | --- | --- | --- | --- | --- | --- | --- | --- | --- |
| hsa04657 | IL-17_signaling_pathway | <http://www.genome.jp/kegg-bin/show_pathway?hsa04657+2919+6374+3576+2354+3934+7128> | 6 | 93 | 31 | 7257 | 2.14153E-06 | 0.000188454 | 5.669276841 | 0.193548387 | CXCL1//CXCL5//CXCL8//FOSB//LCN2//TNFAIP3 |
| hsa04621 | NOD-like_receptor_signaling_pathway | <http://www.genome.jp/kegg-bin/show_pathway?hsa04621+2919+3576+2633+115361+4939+4940+7128> | 7 | 168 | 31 | 7257 | 5.17561E-06 | 0.000227727 | 5.286038636 | 0.225806452 | CXCL1//CXCL8//GBP1//GBP4//OAS2//OAS3//TNFAIP3 |
| hsa05168 | Herpes_simplex_infection | <http://www.genome.jp/kegg-bin/show_pathway?hsa05168+718+23586+3106+64135+3434+4939+4940> | 7 | 185 | 31 | 7257 | 9.782E-06 | 0.000286939 | 5.009572268 | 0.225806452 | C3//DDX58//HLA-B//IFIH1//IFIT1//OAS2//OAS3 |
| hsa05164 | Influenza_A | <http://www.genome.jp/kegg-bin/show_pathway?hsa05164+3576+23586+3383+64135+4939+4940> | 6 | 173 | 31 | 7257 | 7.55016E-05 | 0.001661036 | 4.122043761 | 0.193548387 | CXCL8//DDX58//ICAM1//IFIH1//OAS2//OAS3 |
| hsa04622 | RIG-I-like_receptor_signaling_pathway | <http://www.genome.jp/kegg-bin/show_pathway?hsa04622+3576+23586+64135+9636> | 4 | 70 | 31 | 7257 | 0.000205227 | 0.002892134 | 3.687765666 | 0.129032258 | CXCL8//DDX58//IFIH1//ISG15 |
| hsa05160 | Hepatitis_C | <http://www.genome.jp/kegg-bin/show_pathway?hsa05160+3576+23586+3434+4939+4940> | 5 | 131 | 31 | 7257 | 0.000206928 | 0.002892134 | 3.684181159 | 0.161290323 | CXCL8//DDX58//IFIT1//OAS2//OAS3 |
| hsa05162 | Measles | <http://www.genome.jp/kegg-bin/show_pathway?hsa05162+23586+64135+4939+4940+7128> | 5 | 134 | 31 | 7257 | 0.000230056 | 0.002892134 | 3.638166228 | 0.161290323 | DDX58//IFIH1//OAS2//OAS3//TNFAIP3 |
| hsa05323 | Rheumatoid_arthritis | <http://www.genome.jp/kegg-bin/show_pathway?hsa05323+2919+6374+3576+3383> | 4 | 90 | 31 | 7257 | 0.000538841 | 0.005927249 | 3.268539531 | 0.129032258 | CXCL1//CXCL5//CXCL8//ICAM1 |
| hsa04064 | NF-kappa_B_signaling_pathway | <http://www.genome.jp/kegg-bin/show_pathway?hsa04064+3576+23586+3383+7128> | 4 | 95 | 31 | 7257 | 0.000661425 | 0.006467266 | 3.179519465 | 0.129032258 | CXCL8//DDX58//ICAM1//TNFAIP3 |
| hsa05167 | Kaposi's_sarcoma-associated_herpesvirus_infection | <http://www.genome.jp/kegg-bin/show_pathway?hsa05167+718+2919+3576+3106+3383> | 5 | 173 | 31 | 7257 | 0.000746649 | 0.006570509 | 3.126883647 | 0.161290323 | C3//CXCL1//CXCL8//HLA-B//ICAM1 |
| hsa04668 | TNF_signaling_pathway | <http://www.genome.jp/kegg-bin/show_pathway?hsa04668+2919+6374+3383+7128> | 4 | 108 | 31 | 7257 | 0.001071145 | 0.008569159 | 2.970151764 | 0.129032258 | CXCL1//CXCL5//ICAM1//TNFAIP3 |
| hsa05134 | Legionellosis | <http://www.genome.jp/kegg-bin/show_pathway?hsa05134+718+2919+3576> | 3 | 55 | 31 | 7257 | 0.001593466 | 0.01168542 | 2.79765709 | 0.096774194 | C3//CXCL1//CXCL8 |
| hsa00980 | Metabolism_of_xenobiotics_by_cytochrome_P450 | <http://www.genome.jp/kegg-bin/show_pathway?hsa00980+1645+218+29785> | 3 | 73 | 31 | 7257 | 0.003586607 | 0.024278573 | 2.445316158 | 0.096774194 | AKR1C1//ALDH3A1//CYP2S1 |
| hsa05133 | Pertussis | <http://www.genome.jp/kegg-bin/show_pathway?hsa05133+718+6374+3576> | 3 | 76 | 31 | 7257 | 0.004019054 | 0.025262624 | 2.395876182 | 0.096774194 | C3//CXCL5//CXCL8 |
| hsa04060 | Cytokine-cytokine_receptor_interaction | <http://www.genome.jp/kegg-bin/show_pathway?hsa04060+6368+2919+6374+3576+3575> | 5 | 270 | 31 | 7257 | 0.005274345 | 0.030942826 | 2.277831444 | 0.161290323 | CCL23//CXCL1//CXCL5//CXCL8//IL7R |
| hsa04062 | Chemokine_signaling_pathway | <http://www.genome.jp/kegg-bin/show_pathway?hsa04062+6368+2919+6374+3576> | 4 | 185 | 31 | 7257 | 0.007509193 | 0.041300564 | 2.124406708 | 0.129032258 | CCL23//CXCL1//CXCL5//CXCL8 |
| hsa05169 | Epstein-Barr_virus_infection | <http://www.genome.jp/kegg-bin/show_pathway?hsa05169+23586+3106+3383+7128> | 4 | 200 | 31 | 7257 | 0.009834401 | 0.050907486 | 2.007252097 | 0.129032258 | DDX58//HLA-B//ICAM1//TNFAIP3 |
| hsa05144 | Malaria | <http://www.genome.jp/kegg-bin/show_pathway?hsa05144+3576+3383> | 2 | 49 | 31 | 7257 | 0.018334315 | 0.089634429 | 1.736735312 | 0.064516129 | CXCL8//ICAM1 |
| hsa05161 | Hepatitis_B | <http://www.genome.jp/kegg-bin/show_pathway?hsa05161+3576+23586+64135> | 3 | 144 | 31 | 7257 | 0.022922042 | 0.103708802 | 1.639746692 | 0.096774194 | CXCL8//DDX58//IFIH1 |
| hsa05150 | Staphylococcus_aureus_infection | <http://www.genome.jp/kegg-bin/show_pathway?hsa05150+718+3383> | 2 | 56 | 31 | 7257 | 0.023570182 | 0.103708802 | 1.627637057 | 0.064516129 | C3//ICAM1 |
| hsa05416 | Viral_myocarditis | <http://www.genome.jp/kegg-bin/show_pathway?hsa05416+3106+3383> | 2 | 59 | 31 | 7257 | 0.025981336 | 0.108874169 | 1.585338525 | 0.064516129 | HLA-B//ICAM1 |
| hsa05120 | Epithelial_cell_signaling_in_Helicobacter_pylori_infection | <http://www.genome.jp/kegg-bin/show_pathway?hsa05120+2919+3576> | 2 | 68 | 31 | 7257 | 0.033782247 | 0.13512899 | 1.471311462 | 0.064516129 | CXCL1//CXCL8 |
| hsa05132 | Salmonella_infection | <http://www.genome.jp/kegg-bin/show_pathway?hsa05132+2919+3576> | 2 | 86 | 31 | 7257 | 0.051705902 | 0.19547154 | 1.286459885 | 0.064516129 | CXCL1//CXCL8 |
| hsa05203 | Viral_carcinogenesis | <http://www.genome.jp/kegg-bin/show_pathway?hsa05203+718+3106+5366> | 3 | 201 | 31 | 7257 | 0.05331042 | 0.19547154 | 1.273187895 | 0.096774194 | C3//HLA-B//PMAIP1 |
| hsa05146 | Amoebiasis | <http://www.genome.jp/kegg-bin/show_pathway?hsa05146+2919+3576> | 2 | 96 | 31 | 7257 | 0.062846386 | 0.220832651 | 1.201719694 | 0.064516129 | CXCL1//CXCL8 |
| hsa04933 | AGE-RAGE_signaling_pathway_in_diabetic_complications | <http://www.genome.jp/kegg-bin/show_pathway?hsa04933+3576+3383> | 2 | 99 | 31 | 7257 | 0.066336329 | 0.220832651 | 1.178248565 | 0.064516129 | CXCL8//ICAM1 |
| hsa05142 | Chagas_disease_(American_trypanosomiasis) | <http://www.genome.jp/kegg-bin/show_pathway?hsa05142+718+3576> | 2 | 102 | 31 | 7257 | 0.069890659 | 0.220832651 | 1.155580863 | 0.064516129 | C3//CXCL8 |
