# Supplementary material for: Cancer-associated fibroblast-derived CXCL11 modulates hepatocellular carcinoma cell migration and tumor metastasis through the circUBAP2/miR-4756/IFIT1/3 axis
Source: Cell Death Dis. 2021 Mar 11;12(3):260. doi: 10.1038/s41419-021-03545-7 (PMC7952559; doi:10.1038/s41419-021-03545-7)
Supplement: Supplementary file 7 — supplementary figure and table legends [file 41419_2021_3545_MOESM7_ESM.docx]

**Fig.S1 Selection of chemokines related to HCC development.** Within GSE14323 (A) and GSE6764 (B), CXCL11 expression was also upregulated in liver cirrhosis or liver cancer tissues.

**Fig.S2 CAFs-derived CXCL11 affecting hepatocellular carcinoma (HCC) cell phenotype** HCC cell lines, MHCC-97H and Huh-7, were cultured in control medium (Control), NFs-derived conditioned medium (NFs-CM), CAFs-derived conditioned medium (CAFs-CM), CAFs-CM added with PBS (CAFs-CM/PBS), CAFs-CM added with 5 ng/ml CXCL11 (CAFs-CM/CXCL11 (5 ng/ml)), CAFs-CM added with 10 ng/ml CXCL11 (CAFs-CM/CXCL11 (10 ng/ml)), CAFs-CM added with IgG (CAFs-CM/IgG), and CAFs-CM added with anti-CXCL11 (CAFs-CM/anti-CXCL11). (A) HCC cell DNA synthesis was examined using EDU flowcytometry assay. (B) HCC cell migration was examined using Transwell assay; (C) HCC cell migration was examined by Wound healing assay; (D) The cellular protein content and distribution of Vimentin was examined by IF staining.

**Fig.S3 CAFs-derived CXCL11 affecting the growth of the orthotopically implanted tumor model in BALBc nude mice** (A) Mice were randomly assigned into five groups as described in the Materials and methods section and the orthotopic implantation model was conducted accordingly. Representative images are shown. (B) Fourteen days after cell injection, mice were anesthetized and sacrificed, the livers were collected and the tumor numbers were counted. (C) The histopathological characteristics of livers from different groups were evaluated by H&E staining. (D) (D and E) The protein content and distribution of CXCL11 in liver tumor tissues were examined by Immunoblotting (D) and IHC staining (E). ***P*<0.01, compared with the Huh-7 group; ##*P*<0.01, compared with the Huh-7 + NFs group; &&P<0.01, compared with the Huh-7 + CAFs (sh-CXCL11) group.

**Fig.S4 circUBAP2 affecting hepatocellular carcinoma (HCC) cell phenotype** MHCC-97H and Huh-7 cells were treated with CXCL11 and transfected with si-circUBAP2 and examined for (A) cell migration by Transwell assay; (B) cell migration by Wound healing assay; (C) the cellular protein content and distribution of Vimentin was examined by IF staining;

**Table S1 Primers sequence**

**Table S2 Up-regulation of KEGG pathway table**
